# Entropy-Mediated Lattice Distortion for Tailored Dielectric Polarization to Improve L-Band Electromagnetic Wave Absorption

**DOI:** 10.1007/s40820-026-02203-x

**Published:** 2026-05-07

**Authors:** Han Ding, Yibo Li, Yu Wang, Weikang Song, Xuan Wang, Xijiang Han, Ping Xu, Yunchen Du

**Affiliations:** 1 State Key Laboratory of Space Power-Sources, School of Chemistry and Chemical Engineering, Harbin Institute of Technology, Harbin, 150001 People’s Republic of China; 2 Key Laboratory for Liquid-Solid Structural Evolution and Processing of Materials, Ministry of Education, Shandong University, Jinan, 250061 People’s Republic of China

**Keywords:** Configurational entropy, Lattice distortion, Electromagnetic wave absorption, Dielectric polarization, L-band

## Abstract

Due to the long wavelength of L-band, the precise regulation of electromagnetic (EM) parameters to achieve efficient EM absorption in this frequency range remains a formidable challenge. Here, low-entropy, medium-entropy, and high-entropy (HEA) alloys are designed via a configurational-entropy control strategy, which progressively enhances lattice distortion and reconstructs the electronic structure, thereby enabling precise tuning of real (*ε*_r_*′*) and imaginary parts (*ε*_r_*″*) of the complex permittivity under robust EM loss. Experiments and density functional theory demonstrate that increasing configurational entropy intensifies lattice distortion in multi-principal element alloys, disrupts local central symmetry within the crystal structure, induces an asymmetric reconstruction of the electron cloud, and breaks the degeneracy of electronic states. This process establishes massive dipole polarization centers at the atomic scale. Furthermore, the rugged potential energy surface constructed by this severe distortion imposes a higher energy barrier against the dynamic orientational relaxation of dipoles, which prolongs polarization relaxation time and effectively amplifies the low-frequency dielectric polarization loss. Benefiting from this entropy-driven lattice-distortion effect, the HEA achieves a minimum reflection loss of − 22.7 dB at 1.7 GHz (99.46% absorption efficiency) and an L-band coverage of 80.5% (RL ≤  − 5 dB). 3D gradient multilayer metamaterial structure broadens the bandwidth, achieving absorption of almost 0.5 to 8 GHz (RL ≤  − 10 dB) at 8 mm thickness. Additionally, HEA shows excellent radar stealth and corrosion resistance. This work elucidates the mechanism through which entropy-driven lattice distortion modulates the EM response, providing a novel research paradigm for designing low-frequency EM functional materials via micro-lattice engineering.

**Supplementary Information:**

The online version contains supplementary material available at 10.1007/s40820-026-02203-x.

## Highlights

- This work employs a configurational-entropy control strategy to progressively enhance lattice distortion in multi-principal element alloys, thereby reconstructing their electronic structure and balancing electromagnetic parameters under high electromagnetic loss.
- Density functional theory calculations demonstrate that entropy-induced lattice distortion further drives asymmetric electron cloud reconstruction, thus forming numerous dipolar polarization centers, which enhances low-frequency dielectric attenuation of high-entropy alloy (HEA).
- HEA exhibits excellent low-frequency electromagnetic absorption, radar stealth, and corrosion resistance, while metamaterial structural design further broadens its absorption bandwidth.

**Supplementary Information:**

The online version contains supplementary material available at 10.1007/s40820-026-02203-x.

## Graphical abstract

**Figure:**
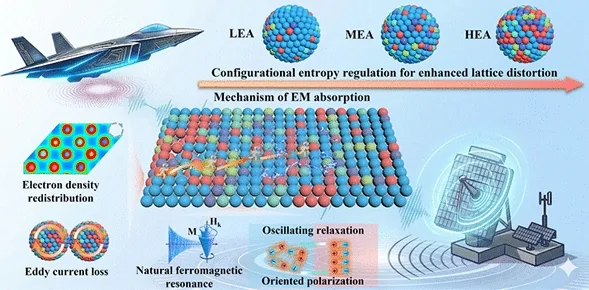

## Introduction

Against the backdrop of widespread deployment in remote sensing radar, global navigation satellite systems (GNSS, typically exemplified by GPS L1 ≈ 1.575 GHz), and electronic warfare systems, the electromagnetic (EM) environment has become markedly more complex, which imposes stricter requirements on high-performance EM wave absorbing materials (EWAMs) for low frequencies (especially the L-band, 1–2 GHz) [1, 2]. The wavelength of the L-band falls within the decimeter-wave range, and thus, theoretically, EWAMs need not only to maintain sufficient magnetic-loss capabilities but also to tune complex permittivity (*ε*_r_) to meet the "quarter-wavelength" design requirement. However, according to the principle of causality (Kramers–Kronig relations) and the conductive channel percolation effect, a significant increase in *ε*_r_*′* of dielectric EWAMs typically accompanies a concurrent increase in *ε*_r_*″*, which leads to a deviation in EWAMs’ input wave impedance from the free space impedance [3]. This results in EM waves being unable to be effectively transferred into the EWAMs coating, causing significant reflection at the surface. To overcome this issue, the design of EWAMs must not only maintain a large *ε*_r_*′* but also precisely control the increase in *ε*_r_*″* to avoid impedance mismatch [4–7]. Therefore, the development of efficient EWAMs for the L-band urgently requires paradigms with programmable microstructures to achieve an appropriate balance between energy storage and dissipation, thereby ensuring optimal impedance matching while maintaining high loss.

In addressing the challenges associated with low-frequency EWAMs, traditional magnetic materials (such as iron, cobalt metals, and carbonyl iron) have long been recognized for their high saturation magnetization and tunable initial permeability [8]. However, their inherent coupling constraints become pronounced in the low-frequency regime: the permeability declines sharply, which impedes sufficient magnetic loss. In addition, limited polarization pathways in single- or binary-element alloys result in weak dielectric loss, and this in turn limits the overall EM energy attenuation capability [9, 10]. Both factors collectively constrain EM absorption in L-band. To overcome these coupling limitations, multi-principal element alloys (MPEAs) differ fundamentally from single- or binary-element alloys. By incorporating multiple metallic components within a single system, MPEAs provide a rich combination of electronic, magnetic, and dielectric functionalities, offering an innovative platform for the concurrent operation of multiple loss mechanisms. Particularly noteworthy is high-entropy alloys, which have since attracted substantial interest since their report by Yeh and colleagues in 2004 [11]. Unlike conventional alloys, high-entropy (HEA) stabilize their microstructures predominantly through high configurational entropy, as opposed to enthalpy-driven interactions. Notably, configurational entropy is not merely a summation of the individual elements. Instead, it induces super-linear synergistic effects at the microscopic level, such as enhancing local chemical coordination differences and generating significant strain fields within the material [12–14]. The contributions of individual metallic elements to EM response are complementary, such that the macroscopic properties of the system often far exceed any single component [15]. Therefore, from the perspective of EM absorption mechanisms, the complementary roles of elements are critical. Magnetic elements such as Fe, Co, and Ni primarily provide tunable magnetic responses and introduce magnetic dissipation channels such as natural resonance and magnetic relaxation. In contrast, elements like Cr, Al, Cu, and Mn tend to enhance dielectric loss by modulating electrical conductivity, defect structures, and the localized charge distribution induced by disorder, which promotes dielectric loss [16–18]. When these elements with diverse physical properties coexist in a high-entropy configuration, they effectively establish multi-scale dielectric-magnetic inhomogeneities, which is the theoretical basis for implementing the “impedance matching–parallel multi-channel loss” strategy required for efficient low-frequency EM absorption.

Furthermore, in high-entropy alloys, configurational entropy acts both as a thermodynamic stabilizing factor and as a “precise regulator” of lattice behavior at the microscopic scale [19–21]. Through atomic-scale strain fields and displacement fluctuations, it profoundly influences electronic structure and energy band distribution, thereby offering a promising pathway for tailoring EM response properties [22–24]. Specifically, the chemical disorder and size/electronegativity disparities associated with high configurational entropy generate significant local strain and lattice distortion. These distortions generate localized stress that disrupt the homogeneity of spatial charge distribution, leading to the formation of macroscopic electric dipole clusters originating from distortion centers, thereby enhancing dielectric relaxation behavior [25, 26]. More importantly, the renormalization of electronic structure caused by these distortions enables reconstruction of the energy band structure and density of states, along with effective regulation of charge carriers [27]. For instance, Liu et al. utilized termination atoms with large ionic radii to induce in-plane strain in the atomic corrugated structure of Nb_2_CTe_x_-based Mxenes, effectively tailoring electronic properties and enhancing polarization relaxation and dielectric loss [25]. In another study, Wen et al. employed a plasma-assisted annealing technique to achieve precise control of lattice compressive strain in MoTe_1.5_S_0.5_, combined with interface engineering to optimize the electronic structure, which ultimately enhanced its high-frequency EM wave absorption performance [28]. Beyond dielectric effects, lattice distortions can influence magnetic configurations and dynamic responses in high-entropy alloys via spin–lattice coupling, thereby regulating the dispersion characteristics of magnetic permeability [29]. This mechanism partially mitigates the limitations imposed by the Snoek's limit on traditional magnetic absorbing materials in the low-frequency range [30]. Thus, lattice distortion serves as a critical bridge connecting atomic-scale structure with macroscopic EM functionality and thus allows synergistic control of dielectric- and magnetic-loss mechanisms. However, the cross-scale correlation mechanism, in which configurational-entropy-driven lattice distortion maps to low-frequency EM parameters through electronic structure reconfiguration, remains insufficiently explored. A deeper understanding of this relationship is essential to drive the development of high-entropy alloys for low-frequency EWAMs applications.

Here, the configurational entropy of MPEAs system (Fe, Co, Ni, Cr, Cu) is rationally regulated by fixing the relative molar ratios of Co, Ni, Cr, and Cu and varying only the Fe content. Fe is selected as the key tuning element because it possesses a stronger intrinsic magnetic moment and higher saturation magnetization than the other transition metals, thereby enabling configurational-entropy modulation while preserving the good magnetic response of the MPEAs. This strategy successfully designs low-entropy (LEA), medium-entropy (MEA), and HEA. As configurational entropy increases, the MPEAs undergo a phase evolution from a BCC-dominated state to an FCC structure, accompanied by more severe lattice distortion, which is quantified by geometric phase analysis (GPA) via the strain distribution. Furthermore, DFT indicates that the pronounced lattice distortion caused by the high-entropy effect in the HEA disrupts the local charge balance, leading to the generation of abundant dipole polarization centers, which enhances dielectric loss and balances *ε*_*r*_*′* and *ε*_*r*_*″*. Simultaneously, the electron spin and lattice coupling suppress the frequency dispersion of the real part of permeability and provides an effective magnetic-loss channel for the HEA. Consequently, the HEA achieves a balance between high loss and impedance matching in the low-frequency L-band, reaches RLmin of -22.7 dB at 1.7 GHz, and exhibits excellent radar-stealth capability. In addition, a three-dimensional metamaterial architecture further expands the effective absorption bandwidth (EAB) to 7.5 GHz (0.5–8.0 GHz, RL ≤ -10 dB). Meanwhile, entropy-induced lattice distortion suppresses Cl^−^ penetration via a hysteretic-diffusion mechanism and promotes Cr/Ni passivation, which improves the corrosion resistance of the HEA. This work elucidates the cross-scale mechanism that links configurational entropy, lattice distortion, and EM functionality, and provides a new route for the design of low-frequency EWAMs.

## Experimental Section

### Materials

Nickel nitrate hexahydrate (Ni(NO_3_)_2_·6H_2_O, purity ≥ 98%) and ferric nitrate nonahydrate (Fe(NO_3_)_3_·9H_2_O, purity ≥ 98.5%) were obtained from Tianjin Damao Chemical Reagent Factory. Cobalt nitrate hexahydrate (Co(NO_3_)_2_·6H_2_O, purity ≥ 99.9%) and chromium nitrate nonahydrate (Cr(NO_3_)_3_·9H_2_O, purity ≥ 99%) were sourced from Shanghai Aladdin Reagent Co., Ltd. Copper nitrate hexahydrate (Cu(NO_3_)_2_·6H_2_O, purity ≥ 99%) and polyacrylic acid (supplied as a 30% aqueous solution) were supplied by Tianjin Guangfu Technology Development Co., Ltd. and Tianjin Komiou Chemical Reagent Co., Ltd., respectively.

### Synthesis of SM, LEA, MEA, and HEA

A single-metal system and three multi-principal element metallic systems with different configurational entropies were successfully synthesized through a polymer-assisted gel method. To prepare the MPEAs, metal nitrates (Fe(NO_3_)_3_·9H_2_O, Co(NO_3_)_2_·6H_2_O, Ni(NO_3_)_2_·6H_2_O, Cu(NO_3_)_2_·3H_2_O, and Cr(NO_3_)_3_·9H_2_O) were precisely weighed with the Fe:Co:Ni:Cr:Cu molar ratios set to 100:0:0:0:0, 85:5.1:5.1:2.6:2.2, 60:13.5:13.5:7.1:5.9, and 35:22:22:11.5:9.5, respectively. Among them, the relative molar ratio of Co, Ni, Cu, and Cr remains unchanged, and the continuous and controllable regulation of the configurational entropy of the alloy system is realized only by adjusting the absolute molar fraction of Fe in MPEAs. The metal salts were added to 20 mL of deionized water and stirred at room temperature until completely dissolved. This solution was then slowly added into a 30 wt% poly acrylic acid (PAA) solution. The resulting mixture was stirred for 24 h to allow the carboxyl groups of PAA to chelate with the metal cations, forming a stable polymer-metal gel. After drying at 70 °C for 24 h, brown solid precursors were obtained, denoted as single-metal precursor (SM-pre), low-entropy alloy precursor (LEA-pre), medium-entropy alloy precursor (MEA-pre), and high-entropy alloy precursor (HEA-pre). Second, each precursor was calcined in a muffle furnace at 700 °C for 6 h to yield the corresponding metal oxides. Finally, the metallic oxides were mixed with CaH_2_ at a mass ratio of 1:3 and ground. The mixture was then thermally reduced in a tubular furnace under an argon atmosphere by heating to 700 °C at a rate of 5 °C min^−1^ and holding for 6 h. After cooling, the products were washed with NH_4_Cl solution to remove CaO and residual CaH_2_, thoroughly rinsed with deionized water, and dried under vacuum at 60 °C. The final samples were designated as single-metal (SM), low-entropy alloy (LEA), medium-entropy alloy (MEA), and high-entropy alloy (HEA). Among them, SM was used as the reference sample.

## Results and Discussion

### Microstructural Characterization of MPEAs

**Fig. 1:**
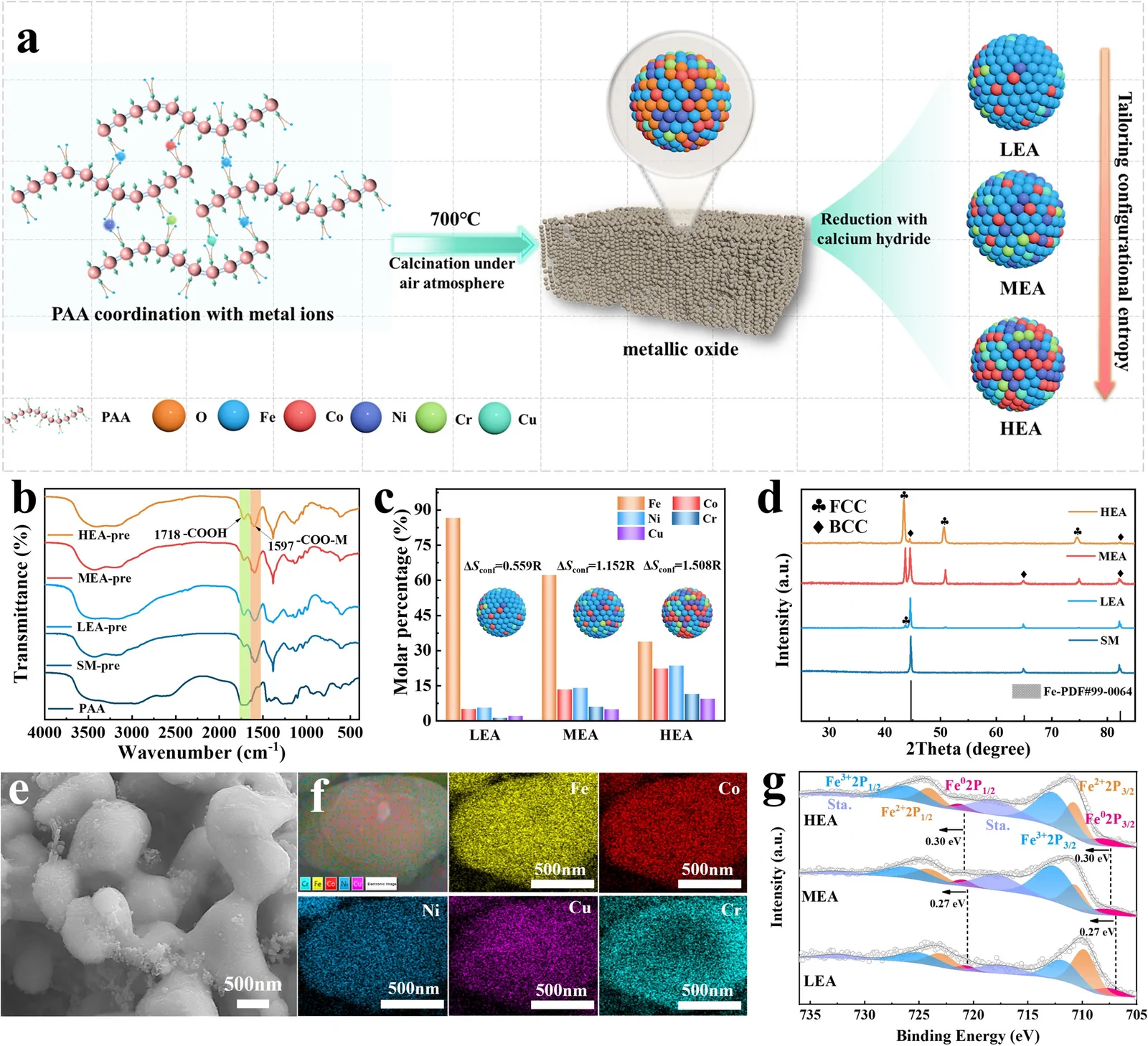
**a** Schematic diagram of the preparation of MPEAs. **b** FTIR spectra of the precursors. **c** ICP-MS analysis of elemental content of LEA, MEA, and HEA. **d** XRD patterns of SM, LEA, MEA, and HEA. **e** FESEM image of the HEA sample. **f** EDS elemental mapping of Fe, Co, Ni, Cu, and Cr in HEA. **g** Fe 2*p* spectra of LEA, MEA, and HEA

Based on a continuous configurational-entropy tuning strategy, a series of MPEAs with low-, medium-, and high-entropy characteristics are successfully synthesized. As illustrated in Fig. 1a, the experimental procedure follows a step-by-step material design route described as “coordination crosslinking-oxidative heat treatment-CaH_2_ mediated reduction”. Initially, a stable ionically cross-linked network is formed through an ion-exchange-driven coordination chemical process, in which deprotonated -COO^−^ groups on poly acrylic acid (PAA) chains complex with positively charged metal ions (M^n+^). Fourier transform infrared spectroscopy (FTIR) analysis provides direct evidence for this coordination, as it shows a new absorption peak at 1597 cm^−1^ that corresponds to the asymmetric stretching vibration of -COO-M (Fig. 1b) [31]. Subsequently, to completely remove the organic template, the resulting precursor is subjected to oxidative heat treatment. The thermogravimetric (TG) curve indicates that mass loss essentially plateaus beyond 500 °C (Fig. S1). Pyrolysis temperature of 700 °C for 6 h is employed to ensure complete removal of the polymer template and carbonaceous residues while promoting metal oxide formation. Finally, alloying is accomplished via a CaH_2_-mediated reduction process. CaH_2_ acts as a reducing agent, decomposing to generate highly reductive active H_2_ and Ca. The standard reduction potential of Ca metal is -2.87 V, which is far lower than the reduction potentials of Fe, Co, Ni, Cr, and Cu metals. Ca spontaneously reduces metal oxides to their metallic states. The high-temperature provides sufficient kinetic energy for atomic migration, enabling metal atoms to overcome diffusion barriers, escape their original lattice sites, and undergo intensive interdiffusion, ultimately forming homogeneous MPEAs phase. During this process, the entropy-increasing effect not only drives significant crystal structure evolution but also induces lattice distortions and defects due to static lattice strain and local chemical bond environment fluctuations caused by atomic size differences. These microstructural changes are anticipated to effectively tailor the electronic properties and influence the EM response mechanisms, which are discussed in subsequent characterization.

To quantitatively reveal changes in molar entropy change (Δ*S*_conf_) at the composition level, inductively coupled plasma mass spectrometry (ICP-MS) is used to precisely determine the molar percentages of Fe, Co, Ni, Cr, and Cu in the three alloys designated LEA, MEA, and HEA (Fig. 1c and Table S1). The calculated Δ*S*_conf_ (Eq. S1) values are 0.559R, 1.152R, and 1.508R, respectively, corresponding to the thermodynamic regimes of typical low-entropy alloys (Δ*S*_conf_ ≤ 1R), medium-entropy alloys (1R < Δ*S*_conf_ ≤ 1.5R), and high-entropy alloys (Δ*S*_conf_ > 1.5R). Therefore, this result has thermodynamically validated the feasibility of continuously tuning entropy through compositional design. X-ray diffraction (XRD) analysis further revealed the regulatory effect of configurational entropy on phase-composition evolution (Fig. 1d and Table S2). The semi-quantitative phase fractions are obtained through background subtraction and peak-area integration of the primary characteristic diffraction peaks for the BCC and FCC phases via MDI Jade 6, followed by normalization against the total integrated area of the characteristic peaks from both phases. The analysis indicate that SM exhibits a pure BCC phase (PDF#99–0064). With increasing configurational entropy, a small amount of FCC phase (8.56%) emerges in the LEA, while the BCC phase (91.44%) remains dominant. In the MEA, the phase fractions converge toward a nearly balanced dual-phase structure (FCC: 52.17%; BCC: 47.83%). For HEA, the FCC phase (92.13%) becomes absolutely predominant, with only a minor residual BCC phase (7.87%), clearly indicating an entropy-driven BCC to FCC transformation. Notably, the (111) diffraction peak of the FCC phase progressively shifts to lower 2θ angles (43.8° → 43.7° → 43.4°) with increasing entropy, reflecting lattice expansion and pronounced distortion, while the diffraction peak position of the BCC phase remains basically stable [32]. This is because BCC is a non-close-packed structure with a large interstitial space inside that can accommodate the mismatch of atomic size, rather than showing a macroscopic lattice expansion [33].

Furthermore, the microstructural features of the HEA, MEA, LEA, and SM samples are observed using field-emission scanning electron microscopy (FESEM). As shown in Fig. 1e, HEA exhibits a three-dimensional interconnected skeletal framework constructed from neck-fused microparticles. During the reduction process, the "in situ foaming" effect from CaH_2_ decomposition generates gases that create initial pores within the HEA, while subsequent removal of CaO by-products generates secondary channels, collectively endowing the HEA with a porous character. Similar structures are observed for the MEA, LEA, and SM, indicating the good generality and structural inheritance of the synthesis method (Fig. S2a-c). Energy-dispersive X-ray spectroscopy (EDS) confirms the uniform distribution of Fe, Co, Ni, Cr, and Cu across HEA, MEA, and LEA samples without any detectable elemental segregation (Figs. 1f and S2d, e). This has confirmed that the entropy-increasing process effectively suppressed the element diffusion segregation dynamics and stabilized the formation of a homogeneous solid solution. Furthermore, the observed single-phase FCC/BCC structure in XRD directly confirms a uniform elemental distribution, which guarantees that the EM properties are intrinsically derived from the configurational-entropy-regulated MPEAs alloy phase, rather than to effects arising from compositional segregation or secondary phases. To further elucidate the evolution of the local electronic environment around Fe with increasing configurational entropy, X-ray photoelectron spectroscopy (XPS) provides key information. The Fe 2*p* fine spectrum shows that the Fe^0^ 2*p* peak shifts toward higher binding energy as configurational entropy increases. Specifically, the shift is 0.27 eV from LEA to MEA and a further shift of 0.3 eV from MEA to HEA (Fig. 1g). The shift in binding energy reflects changes in the electron cloud density around the Fe atoms [34, 35]. This indicates that compositional fluctuations in the MPEAs reconstruct the electronic environment around Fe atoms, induce the formation of local dipolar polarization centers, and thereby facilitate the optimization of the EM parameters.

**Fig. 2:**
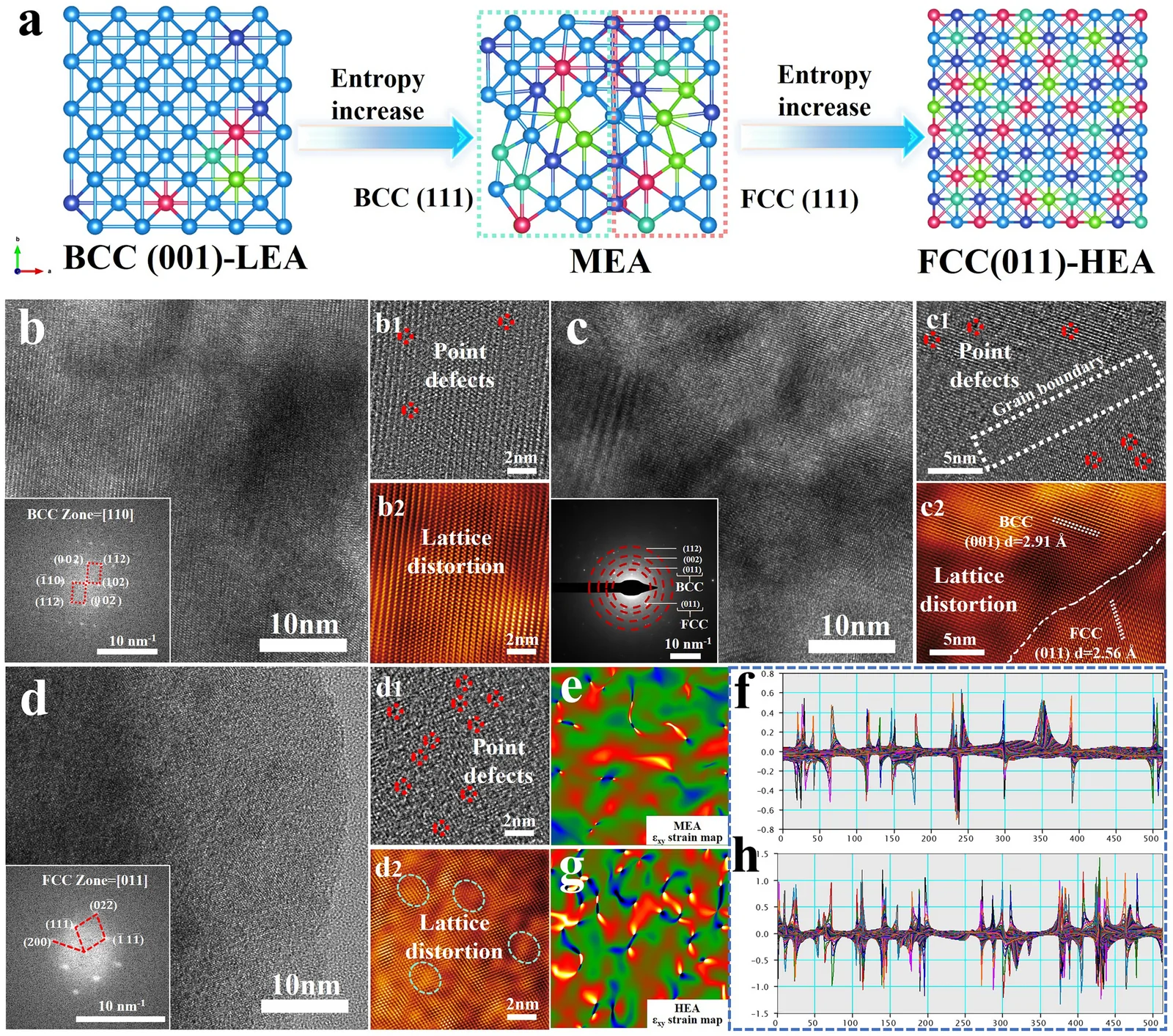
**a** Ideal atomic models of LEA, MEA, and HEA. HR-TEM images of **b** LEA, **c** MEA, and **d** HEA reveal the crystal structure details (insets show the corresponding FFT patterns (b₁-d₁ and b₂-d₂). GPA strain map and the corresponding local strain intensity curve for **e, f** MEA and **g, h** HEA

To gain in-depth insights into the impact of configurational-entropy variation-driven crystal phase evolution on the microstructure of MPEAs, this study establishes ideal atomic models for LEA, MEA, and HEA (Fig. 2a), which visually illustrate the transition process of the crystal phase from BCC to FCC. The model shows that as entropy increases, the atomic distribution progressively evolves from the BCC structure in low-entropy LEA to a mixed phase with partially embedded FCC structure in MEA, and finally forms a highly disordered FCC lattice in HEA, which intuitively reflects the trend of high-entropy effect on crystal structure regulation. Based on this model, we further conduct systematic characterization of the samples using aberration-corrected high-resolution transmission electron microscopy (HR-TEM) and selected-area electron diffraction (SAED). In Fig. 2b, the red dotted box auxiliary indicates the arrangement correlation of some diffraction spots, which follow the diffraction rules of BCC along the [110] zone axis, indicating that the LEA sample is mainly composed of BCC phase. For the MEA (Fig. 2c), calibration of the polycrystalline diffraction rings reveals the coexistence of BCC and FCC phases. The fast Fourier transform (FFT) image shown in Fig. 2c2 further indicates that the increase in configurational entropy promotes phase separation and leads to the formation of distinct phase interfaces. The measured interplanar spacings of 2.91 and 2.56 Å correspond to the (001) plane of BCC and the (011) plane of FCC, respectively. Upon increasing entropy to the high-entropy state, the crystal phase of MPEAs undergoes a further transition toward the FCC structure. The electron diffraction pattern aligns with the diffraction symmetry of an FCC crystal along the [011] zone axis, confirming that the HEA sample is predominantly composed of the FCC phase (Fig. 2d). It should be noted that during the progressive increase in configurational entropy, FFT images reveal that the HEA exhibits more pronounced variations in interatomic spacing across many regions compared to LEA and MEA, and intensifies severe lattice distortion (Fig. 2a2–c2). To quantitatively evaluate this effect, the TEM-based geometric phase analysis (GPA) is employed to analyze the influence of configurational entropy on lattice distortion. Under the same analysis conditions, the strain mapping of HEA shows a broader distribution of tensile and compressive strains compared to LEA and MEA (Figs. 2e–g and S3a). Furthermore, a quantitative verification of this phenomenon was performed by combining the linear profile of strain magnitude and statistical metrics: the linear profile reveals that the fluctuation amplitude and absolute extreme values of the ε_xy_ strain are larger for HEA (Figs. 2f-h and S3b). The standard deviation of ε_xy_ increases monotonically from LEA to MEA to HEA, indicating that the strain field dispersion continues to increase and the strain distribution becomes more inhomogeneous, which implies that lattice distortion becomes more widespread and severe over a larger spatial range (Fig. S4a-c). These results collectively validate that the increase in configurational entropy effectively enhances lattice distortion in the MPEAs. This distortion primarily arises from atomic size mismatch and chemical disorder induced local lattice strain, which significantly disrupts the periodicity of the lattice and the uniformity of the elastic field. Additionally, during the entropy increase process, varying degrees of defect sites are observed in Fig. 2b1–d1. These defects act as additional sources of lattice perturbation and couple with atomic size mismatch strain, which further amplifies the lattice distortion, thereby altering the electronic properties of the material and modulating its EM performance.

### Role of Entropy-Driven Lattice Distortion in Low-Frequency EM Absorption

**Fig. 3:**
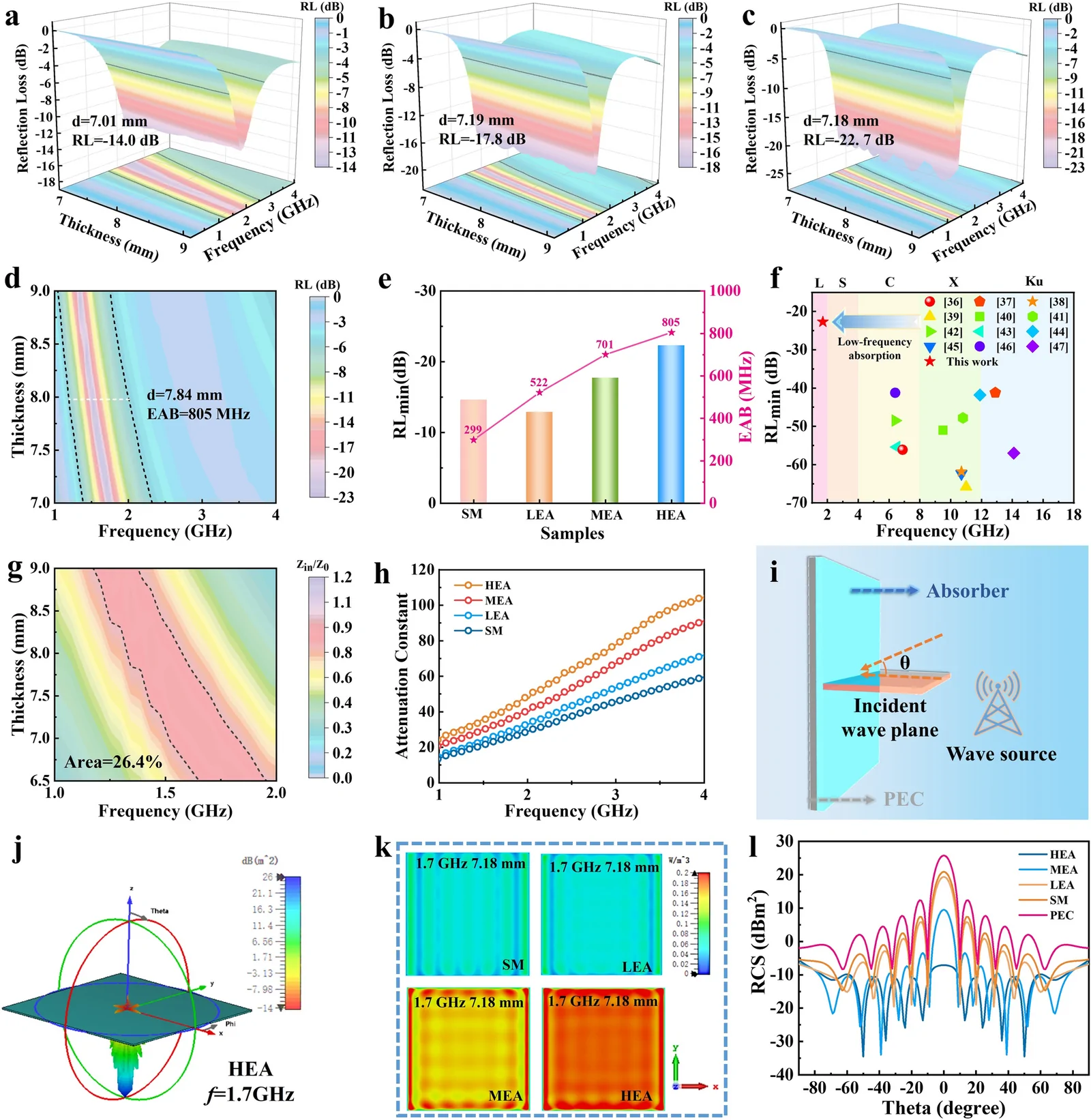
3D diagrams of the reflection loss curve of **a** LEA, **b** MEA, and **c** HEA. **d** 2D contour plots of reflection loss for HEA. **e** Comparison of RLmin and EAB for all samples. **f** Comparison of absorption frequency ranges of HEA versus reported high-entropy absorbers. **g** *Z*_in_/*Z*_0_ values for SM, LEA, MEA, and HEA. **h** *α* as a function of frequency for SM, LEA, MEA, and HEA. **i** Schematic diagram of the CST simulation model. **j** 3D RCS plots for the PEC substrate covered with HEA. **k** Simulated RCS curves of the PEC substrate and SM, LEA, MEA, and HEA with varying incidence angles. **l** Power loss density for SM, LEA, MEA, and HEA

To validate the effect of lattice distortion induced by increasing entropy on the EM wave absorption capability, the RL values of SM, LEA, MEA, and HEA are calculated using transmission line theory to evaluate the EM absorption performance (Eqs. S2 and S3). As shown in the 3D reflection loss contour maps (Figs. 3a–c and S5a), HEA exhibits superior EM absorption performance compared to SM, LEA, and MEA, achieving a minimum reflection loss (RL_min_) of -22.7 dB at 1.7 GHz (corresponding to an absorption efficiency of 99.46%) with a matching thickness of 7.18 mm. To visually demonstrate the enhancement of low-frequency EM absorption performance by entropy-induced lattice distortion, this study further compares the EM absorption performance of SM, LEA, MEA, and HEA in the L-band (1–2 GHz) at a fixed thickness of 7.84 mm. As illustrated in Fig. 3d, e, HEA demonstrates exceptional low-frequency absorption characteristics at this thickness, with RLmin of -21.3 dB (absorption rate approximately 99.3%) and the EAB (defined as RL ≤ -5 dB, representing over 68.4% absorption) reaching 805 MHz (1.195–2.0 GHz), which accounts for 80.5% of the L-band. Additionally, EAB of 588 MHz is achieved at RL ≤ -7 dB (over 80% absorption). In contrast, SM, LEA, and MEA cover ranges of 299, 522, and 701 MHz (RL ≤ -5 dB) in the L-band, with RLmin values also lower than that of HEA. Compared to existing high-entropy alloy absorbers, which were predominantly concentrated in the C, X, and Ku bands, this work has achieved 80.5% coverage in the low-frequency L-band, which demonstrates excellent low-frequency absorption performance (Fig. 3f and Table S3) [36–47]. Further analysis is conducted on two critical parameters determining RLmin: impedance matching (the closer |*Z*_in_/*Z*_0_| is to 1, the better the impedance matching performance) and the attenuation constant (α) ((Figs. 3g, h and S5b-d, Eqs. S4 and S5) [48, 49]. To quantitatively assess the improvement in impedance matching, the area of the region where 0.8 ≤|*Z*_in_/*Z*_0_|≤ 1.2 is introduced as a measure of the impedance matching window size for EM wave penetration in the L-band [50]. The results show that the impedance matching areas for SM, LEA, MEA, and HEA are 18.5%, 27.6%, 38.7%, and 26.4%, respectively, indicating that MPEAs significantly expand the matching region in the low-frequency range. It should be noted that impedance matching is a necessary but not sufficient condition for strong EM absorption. Although MEA has the largest impedance matching area, HEA still exhibits the best EM absorption performance, primarily due to its highest α in the L-band, while maintaining sufficient impedance matching. This allows EM waves entering HEA to achieve more efficient EM energy dissipation. This result reflects the better balance achieved by HEA between impedance matching and attenuation capability. This characteristic originates from entropy-induced lattice distortion within HEA, which optimizes the dielectric response of HEA, enhancing its low-frequency EM absorption capability. The underlying mechanisms of EM absorption are described in detail later. These results have underscored the critical role of configuration entropy in regulating lattice distortion to enhance low-frequency EM absorption.

To evaluate the stealth performance of the samples in electronic warfare, 3D radar cross-section (RCS) simulations are performed using CST Microwave Studio [51–53]. The simulations model structures with an ideal perfect electric conductor (PEC) panel that is coated with SM, LEA, MEA, and HEA composites, respectively, to quantitatively analyze their EM wave dissipation capability under far-field conditions (Fig. 3i). The 3D RCS results indicate that the PEC panel coated with HEA exhibits lower reflection signals in the primary scattering direction compared to the other samples, which demonstrates its superior EM absorption capability (Figs. 3j and S6a-d). Additionally, the EM energy loss distribution shows that HEA exhibits stronger EM dissipation capability in the low-frequency region (Fig. 3k). Further analysis of the 2D RCS angular distribution from -90° to 90° reveals that the HEA coating achieved a peak RCS value of -34.51 dB m^2^, while it maintains RCS levels below -10 dB m^2^ over nearly the entire angular detection range (Fig. 3l). This performance highlights its exceptional wide-angle stealth characteristics. This simulation result aligns well with the measured RL trends of the actual samples, which further validates its potential for practical low-frequency EM stealth applications.

**Fig. 4:**
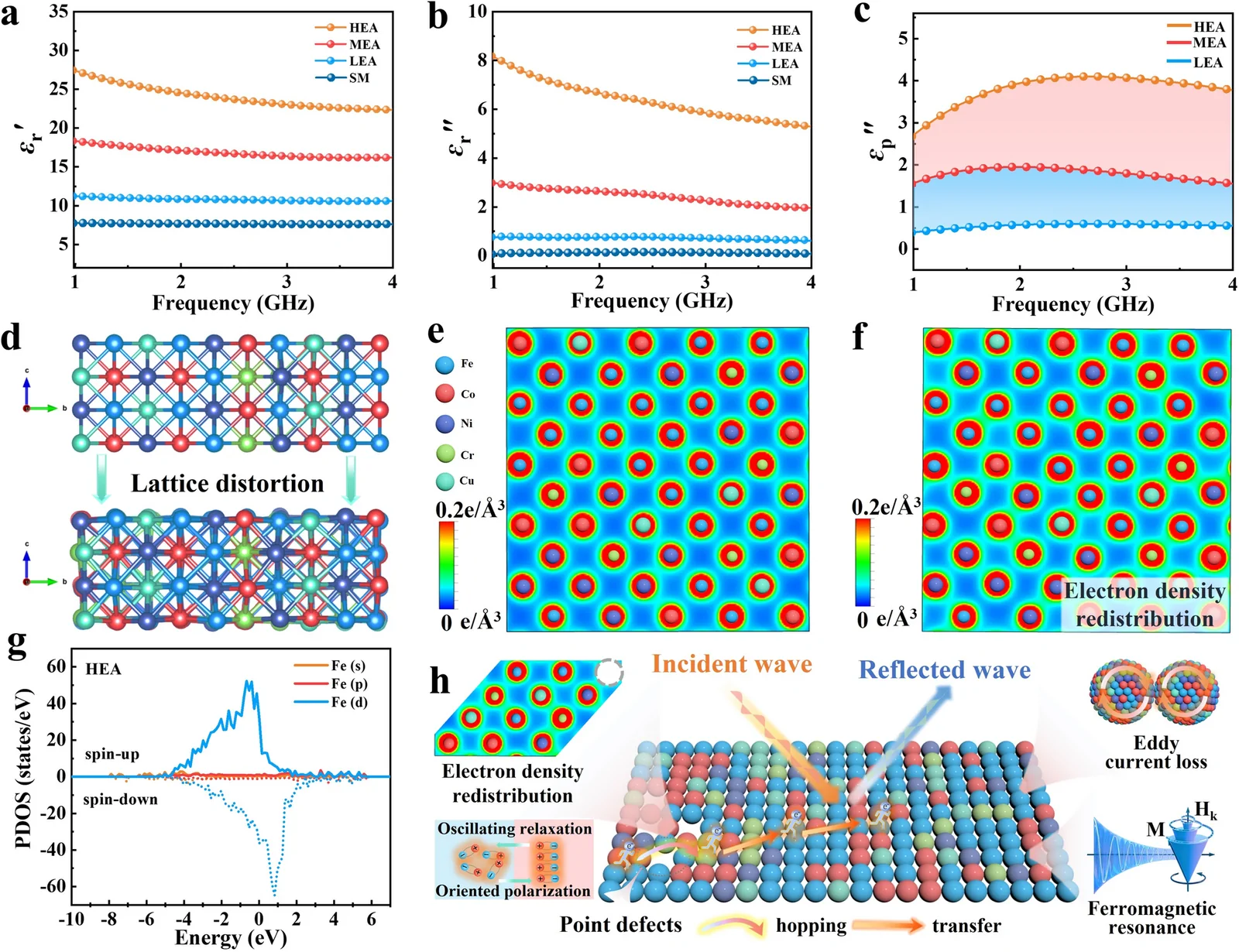
**a** Real permittivity of SM, LEA, MEA, and HEA. **b** Imaginary permittivity of SM, LEA, MEA, and HEA. **c** Comparison of the polarization loss intensity for LEA, MEA, and HEA. **d** MPEAs models in distortion-free and lattice-distorted states. **e, f** Charge density mapping for MPEAs models in distortion-free and lattice-distorted states. **g** Spin projected density of states (PDOS) based on the ideal model of HEA. **h** Mechanism of EM absorption driven by entropy-induced lattice distortion

The L-band EM waves, with their decimeter wavelength, require EWAMs to optimize impedance matching and extend the interaction range by increasing the matching thickness for efficient absorption, while also imposing stringent demands on the low-frequency adaptability and tunability of their EM parameters. In this context, the role of entropy-driven lattice distortion in low-frequency EM response is further elucidated through the modulation of EM parameters. As shown in Fig. 4a, *ε*_r_*′* exhibits a significant stepwise increase with increasing configurational entropy, progressing through SM, LEA, MEA, HEA. Among these, HEA maintains the highest *ε*_r_*′* value throughout the 1–4 GHz frequency band, which demonstrates exceptional electric field energy storage and response capabilities [54, 55]. This enhancement is attributed to the increase in configurational entropy driven by the introduction of various atomic-scale components, which induces severe lattice distortion, disrupts the long-range order of the atomic arrangement, and establishes numerous non-centrosymmetric local structures. This leads to the creation of numerous local dipoles and thereby effectively strengthens the overall energy storage capability [24]. Further analysis of *ε*_r_*″*, the key parameter characterizing dielectric energy loss, reveals a similar upward trend with increasing entropy (Fig. 4b). Notably, the HEA exhibits not only the most robust dielectric loss capability but also a more pronounced low-frequency dielectric dispersion relative to the other samples. In accordance with the classical Debye dielectric relaxation theory, this behavior indicates a significantly prolonged polarization relaxation time (*τ*) within the HEA. On the microscopic physical loss mechanism level, the evolution of *ε*_r_*″* is controlled by the competition and synergy between polarization loss and conduction loss [56, 57]. This phenomenon is clearly distinguished by least squares curve fitting to differentiate the contributions of different loss mechanisms (Fig. S7a-d; Eqs. S6-S9). It is observed that in the 1–1.5 GHz frequency range, conduction loss dominated in HEA, but as the frequency increased, polarization loss gradually becomes the dominant mechanism. For MEA, LEA, and SM, polarization loss exceeds conductive loss across the entire frequency range. To quantitatively elucidate the contribution of entropy-induced lattice distortion to polarization loss, the integral area of the polarization loss component (*ε*_p_*ʺ*) in HEA’s *ε*_r_*ʺ* is calculated and compared with MEA and LEA. The results fully prove that the HEA with severe lattice distortion exhibits a significantly amplified polarization loss. This occurs because, during the entropy increase process, the increase in alloy composition fluctuations (namely, a decrease in Fe and an increase in multicomponent elements) causes the electronic structure to evolve from a “Fe-dominated localized state” to a “multi-element hybridized delocalized state” (Fig. S8a-c) [58]. This electron delocalization is accompanied by severe lattice distortion. This synergistic effect not only induces local charge fluctuations to generate abundant permanent dipole moments but also reconstructs a highly disordered local potential energy landscape, and thereby elevates the energy barrier for dipole reorientation. The positive impact of lattice distortion on dielectric loss is further validated through charge density distributions obtained from DFT calculations. An intuitive comparison between the chemically disordered model without explicit distortion and the actual lattice-distorted model was made by analyzing the charge density profiles of the (001) crystal plane in MPEAs (Fig. 4d–f). In the ideal distortion-free state, although differences in electronegativity and valence-electron concentration among Fe, Co, Ni, Cr, and Cu induce a preliminary rearrangement of the electron cloud, the lattice maintains high translational and central symmetry, with each atom occupying an ideal lattice point. The spatial overlap of positive and negative charge centers results in negligible intrinsic dipole moments, a condition that fundamentally constrains dipole polarization loss. In contrast, the lattice distortion induced by entropy increases results in the formation of multitude of non-centrosymmetric local structures. The localized compression or expansion of atomic positions alters bond lengths and bond angles, elevates the local potential energy, and perturbs the atomic orbital overlap. These effects break the local inversion symmetry within the lattice. Consequently, the coupled distortion of the geometric and electronic structures drives an asymmetric reconstruction of the electron cloud. The charge density sections reveal a displacement of the positive and negative charge centers, thereby constructing local dipole centers at the atomic scale. Under alternating EM field excitation, these distortion-enhanced local dipoles must overcome a higher local potential barrier to complete orientational polarization in response to the external electric field. This effect extends the polarization relaxation time and significantly enhances the EM energy dissipation during the dynamic reorientation of dipoles [25, 59–61]. Thanks to this, the low-frequency dielectric polarization loss performance of HEA is further enhanced. Moreover, HR-TEM images reveal that lattice distortion increases the energy barrier for atomic migration, which reduces the likelihood of atoms migrating to lattice vacancies or defect sites. As entropy increases, more defects form in the lattice, and these defects alter the symmetry of the charge distribution, which in turn triggers polarization relaxation, leading to an increase in polarization loss. Additionally, as entropy increases, the growing proportion of high-concentration free-electron metals in the MPEAs raises the free-electron concentration, which facilitates electron migration under an external electric field and thereby enhances conductive loss. Analysis of dielectric loss tangent (tan*δ*_*ε*_) confirms HEA's superior dissipation capability, with values maintained at 0.27–0.3 (Fig. S9a). According to the established literature, a tan*δ*_*ε*_ value in the range of 0.25–0.5, combined with high *ε*_r_*′* + *ε*_r_*″* values, optimally facilitates low-frequency EM absorption [62]. This suggests that the *ε*_r_*′* of HEA provides sufficient energy storage, while the precise control of the growth of *ε*_r_*″* ensures impedance matching under efficient energy dissipation conditions. The synergistic effect of these two factors forms the foundation for strong low-frequency EM absorption.

The regulatory effect of configurational-entropy variation on the magnetic properties of MPEAs is systematically investigated by vibrating sample magnetometer (VSM) characterization and further combined with DFT calculations of the Fe 3d orbital projected density of states (PDOS) to reveal the mechanism of the high-entropy effect on the microscopic magnetism and electronic structure. The experimental results demonstrate a progressive decrease in saturation magnetization (*M*s) with increasing configurational entropy, where the HEA exhibits a value of 65.3 emu g^−1^ (Fig. S9b). From the perspective of crystal structure, the increase in configurational entropy drives the HEA toward an FCC-dominated phase. Given that *M*s of FCC lattice in MPEAs is intrinsically lower than that of its BCC structure, this entropy-driven phase transformation results in the weakened magnetism of HEA. Moreover, DFT analysis reveals that HEA displays a reduced Fe 3*d* density of states accompanied by weakened spin asymmetry, whereas MEA and LEA maintain higher density of states with enhanced spin asymmetry (Figs. 4g and S9c, d). This is because the multi-element mixing in HEA redistributes the constituent PDOS, which diminishes the Fe 3*d* electron density and generates weaker local magnetic moments. Consequently, this weakens the overall static magnetism, leading to reduced permeability and magnetic-loss tangent (Fig. S9e-g; Eqs. S10 and S11). Nevertheless, this attenuation of magnetism does not equate to the failure of magnetic-loss mechanisms. Specifically, the weakening of localized magnetic moments is accompanied by enhanced electron spin scattering and localized magnetic perturbations. This significantly increases magnetic damping and effectively suppresses the severe frequency dispersion of the real part of permeability (*µ*_r_*′*) in the low-frequency band (Fig. S9h). This mechanism thereby secures a stable magnetic-loss channel for the HEA. To further distinguish the sources of magnetic loss in MPEAs in the low-frequency range, the eddy current coefficient (*C*_*0*_) is introduced to discriminate between different magnetic-loss channels. In the GHz frequency range, the magnetic loss of ferromagnetic materials primarily originates from eddy current loss and natural ferromagnetic resonance [63–65]. Eddy current loss is typically associated with induced circulating currents within EWAMs, and it is characterized by a *C*_0_ value that remains constant within a specific frequency range, not fluctuating with frequency. Calculations of *C*_0_ (Eq. S12) reveal that SM and LEA exhibit a continuous decrease in *C*_0_ across the 1–4 GHz frequency range, without showing an obvious platform region, indicating that their magnetic-loss mechanism is primarily governed by natural ferromagnetic resonance (Fig. S10a, b). This phenomenon occurs because the strong frequency dispersion effect generated during the precession of magnetic moments overcoming the anisotropy field masks the weak eddy current contribution [66]. In the 1–2 GHz range, the *C*_0_ values of MEA show a gradual decrease, further revealing the dominance of natural ferromagnetic resonance. However, in the 2–4 GHz range, the *C*_0_ curve flattens with slight fluctuations, suggesting that eddy current loss begins to contribute, working in tandem with the residual ferromagnetic resonance loss (Fig. S10c). For HEA, the smooth decline of the *C*_0_ curve in the 1–2 GHz range indicates an increasing proportion of eddy current loss, while its flattening in the 2–4 GHz range indicates that the magnetic-loss mechanism is primarily governed by eddy current loss (Fig. S10d). This phenomenon arises from the atomic radius differences of elements in HEA, which lead to a non-uniform distribution of internal magnetic anisotropy fields, weakening the ferromagnetic resonance intensity and enhancing the contribution of eddy current loss. Moreover, in the 1–4 GHz frequency range, we calculated the percentage contribution of tan*δ*_*ε*_ and tan*δ*_*μ*_ to the total loss tangent (Fig. S10e, f). The results show that as configurational entropy increases, the contribution of magnetic loss gradually decreases, while the contribution of dielectric loss increases. This is because the severe lattice distortion induced by the high-entropy state not only reconstructs the magnetic-loss mechanism but also provides favorable conditions for polarization relaxation. Figure 4h details the impact of entropy-induced lattice distortion on the EM wave absorption mechanisms. Regarding dielectric loss, the lattice distortion in HEA disrupts long-range ordering and generates numerous localized dipolar polarization centers at the microscopic scale. Under the excitation of EM waves, these centers sustain dynamic alternations of orientational polarization and irregular thermal motions, which provides an effective channel for low-frequency dielectric loss. Additionally, the migration of free electrons in the high-entropy alloy further enhances conduction loss and significantly amplifies the efficiency of low-frequency dielectric dissipation. In terms of magnetic loss, with increasing configurational entropy, lattice distortion in HEA significantly disrupts the electronic structure, weakening the localized magnetic moments and affecting the magnetic anisotropy field. This change suppresses the contribution of natural ferromagnetic resonance, while the proportion of eddy current losses gradually increases. Therefore, the lattice distortion induced by entropy increase modulates the electronic structure, which enables a dynamic regulation between dielectric and magnetic loss, consequently achieving stable and efficient EM energy dissipation channels in the low-frequency band. This has provided microscopic guidance for the design of high-entropy alloy-based EWAMs.

### Design of the Metamaterial Absorbers

MPEAs exhibit excellent EM absorption potential due to the significant lattice distortion induced by their high-entropy effect. However, even with good intrinsic loss mechanisms, MPEAs in conventional 2D planar structure cannot overcome the bandwidth-thickness trade-off dictated by the Planck–Rozanov limit, nor can they mitigate performance degradation that results from sensitivity to incident angles and polarization states [67, 68]. Therefore, relying solely on traditional 2D planar structure has certain limitations in practical applications. In this context, the introduction of 3D metamaterial structure breaks through these limitations: On the one hand, 3D metamaterial structure exhibits much less degradation in EM absorption performance with varying incident angles and polarization states compared to 2D planar structure; on the other hand, the combination of gradient impedance structure and multiple resonance modes can synergistically broaden the effective EM absorption bandwidth and achieve good matching with free space [69]. Therefore, the deep integration of MPEAs with 3D metamaterial structure not only enhances the EM loss but also effectively broadens EAB, making it highly promising for designing high-performance EWAMs.

**Fig. 5:**
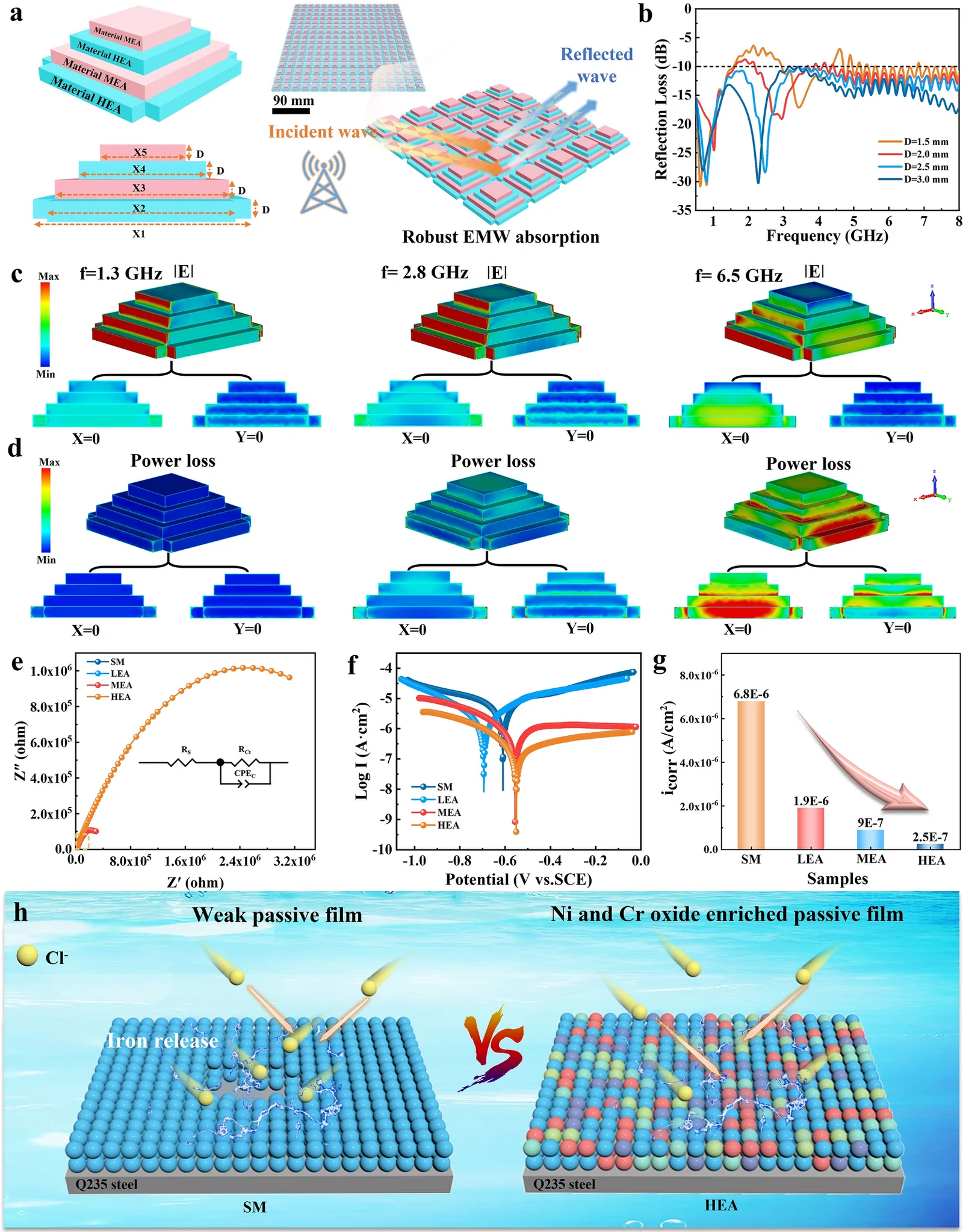
**a** Schematic diagram of the proposed metamaterial EM wave absorber and structural parameters. **b** RL of the metamaterial absorber at different thicknesses. **c, d** Distribution of the electric field and power loss density at 1.3 GHz, 2.8 GHz, and 6.5 GHz for the metamaterial absorber. **e** Nyquist plots of SM, LEA, MEA, and HEA. **f** Tafel plots recorded in 3.5 wt% NaCl solution for SM, LEA, MEA, and HEA. **g** Comparison of corrosion current density for SM, LEA, MEA, and HEA. **h** Diagram illustrating the corrosion protection mechanism

To overcome these limitations, this study has designed a multilayer metamaterial structure based on a four-layer periodic unit cell, with overall dimensions of 360 mm × 360 mm, which incorporates a periodic square-hole array at its bottom-most layer (Fig. 5a). The overall unit cell is defined by six parameters: five horizontal parameters (X1-X5) and one vertical parameter (D). Subsequently, an optimized filling strategy that alternates MEA and HEA in each unit layer constructs a composite architecture with gradient impedance characteristics and multiple resonance responses. This approach has established a structural foundation to broaden the EAB of MPEAs. Analysis of the RL curve indicates that by tuning the unit layer thickness parameter D, this metamaterial exhibits excellent EM absorption performance across the 0.5–8 GHz range (Fig. 5b). When D = 2 mm, its EAB covers multiple bands including P, L, S, and C bands. The RL values are nearly all below -10 dB across the entire frequency range, with distinct absorption peaks appearing in each band. This broadband EM absorption performance primarily originates from multi-frequency local resonances excited by the synergistic effect of the gradient layered structure and the bottom-layer periodic square-hole array. To gain deeper insight into the underlying mechanisms of EM energy dissipation, we analyze the EM field and power loss distributions of the 3D metamaterial structure at three characteristic frequencies (1.3, 2.8, and 6.5 GHz) when D = 2 mm (Figs. 5c, d and S11). At the low frequency of 1.3 GHz, the electric field concentrates primarily at the bottom layer and the corners of the units, while the magnetic field distributes around the inter-layer corners and the square-hole regions. The coupling between the electric and magnetic fields is relatively weak, resulting in comparatively lower power loss density. At 2.8 GHz, the spatial distribution pattern of the EM fields is similar to that at 1.3 GHz; however, the field intensities are enhanced across all these regions. This enhancement promotes both dielectric and magnetic losses, thereby increasing the overall EM energy dissipation efficiency. At 6.5 GHz, the EM fields become highly concentrated at the interfacial regions between the middle and lower layers, forming strong field localization. This indicates significant localized electric and magnetic resonance coupling, which corresponds well with the power loss regions and further demonstrates efficient dissipation of EM energy. These results collectively indicate that the combination of the four-layer periodic gradient structure with the bottom square-hole array not only enhances the local resonance effect but also effectively strengthens EM field coupling. This design has successfully achieved highly efficient EM absorption across a wide frequency band.

### Corrosion Resistance of MPEAs

Given the widespread application of EWAMs in harsh environments such as naval vessel stealth and offshore platforms, their service conditions impose stringent requirements on corrosion resistance. To evaluate the potential of the fabricated MPEAs coatings as long-term corrosion-resistant EWAMs, we systematically investigate their corrosion behavior in a 3.5 wt% NaCl solution (simulating seawater conditions) using electrochemical measurement techniques. Electrochemical impedance spectroscopy (EIS) analysis reveals that the HEA sample has exhibited the largest capacitive arc radius in the Nyquist plot, with a significantly higher charge transfer resistance (*R*_ct_) compared to other samples (Figs. 5e and S12). This indicates strong suppression of corrosion reactions at the HEA surface, where the surface barrier effectively impedes the penetration of the impedance medium and charge transfer, thus reducing sensitivity to corrosion. This is primarily due to the increased surface resistance of the HEA, which forms a denser oxide film more readily than the SM [70]. Subsequently, Tafel polarization curve measurements further corroborate the superior corrosion resistance of the HEA (Fig. 5f). The HEA exhibits a more positive corrosion potential (*E*_corr_) of -0.551 V, suggesting the lowest thermodynamic tendency for corrosion initiation. Quantitative analysis of corrosion current density (*i*_corr_) shows a monotonic decrease in icorr with increasing configurational entropy (Fig. 5g). The icorr of the HEA coating is as low as 2.5 × 10^–7^ A cm^−2^, which is nearly an order of magnitude lower than that of SM and confirms its slowest corrosion rate from a kinetic perspective. Before the passivation zone, the anode curve of the HEA shows a broad and stable passivation region with a relatively stable current density, which suggests that its highly stable passivation film can effectively resist Cl^−^ breakdown and inhibit metal activation dissolution. Figure 5h further elucidates the corrosion resistance mechanism of MPEAs. In the SM coating, where atomic mobility is relatively high, elements tend to diffuse and oxidize rapidly but unevenly, which leads to the formation of a defective “weak passivation film” that cannot effectively block the inward diffusion of corrosive Cl^−^ and results in continuous Fe dissolution at the film/substrate interface. Conversely, the lattice distortion in HEA extends the diffusion path of Cl^−^, increasing the resistance to ion penetration into the substrate. Furthermore, as configurational entropy increases, the “driving force” for atomic diffusion decreases, while lattice distortion increases the “energy barrier” for atomic diffusion. This “hysteresis diffusion effect” together slows down the diffusion rate of Cl^−^ in the HEA [71]. Consequently, the oxidation of passive elements such as Cr and Ni no longer remains dominated by rapid diffusion. Instead, it proceeds along a more thermodynamically selective route. This behavior promotes the formation of a protective passive film with a denser structure and a lower defect density on the surface, thereby effectively delaying corrosion of the substrate. The HEA that integrates exceptional EM absorption performance with superior corrosion resistance has provided new insights for designing durable EWAMs suitable for extreme marine environments.

## Conclusions

In summary, a continuous configurational-entropy control strategy produces MPEA systems spanning LEA to HEA. As configurational entropy increases, the MPEAs undergo a phase evolution from a BCC-dominated state to an FCC structure, and in the high-entropy state atomic-size mismatch and chemical disorder induce pronounced lattice distortion. DFT calculations confirm that lattice distortion further modulates the electronic structure, disrupts uniform charge distribution, and generates numerous dipole polarization centers, which gives the HEA superior dielectric loss relative to the MEA and LEA. Benefiting from entropy-induced lattice distortion, the HEA balances high loss with impedance matching in the low-frequency regime and achieved the RLmin of -22.7 dB at 1.7 GHz in the L-band. RCS simulations further verify excellent wide-angle radar-stealth performance. Moreover, an alternately stacked MEA/HEA metamaterial architecture expands the effective absorption bandwidth to 7.5 GHz (0.5–8.0 GHz, RL ≤ -10 dB). The HEA also exhibits outstanding corrosion resistance and thereby demonstrates strong suitability for harsh environments. For low-frequency broadband EM absorption, entropy-driven lattice distortion serves as a programmable microscopic knob in combination with 3D gradient metamaterial structures. This approach enables optimization of low-frequency broadband EM absorption, which has opened a new path for developing next-generation, material-structure–function integrated EWAMs.

## Supplementary Information

Below is the link to the electronic supplementary material.

Supplementary file1 (DOCX 4590 KB)
