# Supplementary material for: Entropy-Mediated Lattice Distortion for Tailored Dielectric Polarization to Improve L-Band Electromagnetic Wave Absorption
Source: Nanomicro Lett. 2026 May 7;18:358. doi: 10.1007/s40820-026-02203-x (PMC13153311; doi:10.1007/s40820-026-02203-x)
Supplement: Supplementary file 1 — Supplementary file1 (DOCX 4590 KB) [file 40820_2026_2203_MOESM1_ESM.docx]

Supporting Information

**Entropy-Mediated Lattice Distortion for Tailored Dielectric Polarization to Improve L-Band Electromagnetic Wave Absorption**

Han Ding^1^, Yibo Li^2^, Yu Wang^1^, Weikang Song^1^, Xuan Wang^1^, Xijiang Han^1^, Ping Xu^1^ and Yunchen Du^1^*

^1^ State Key Laboratory of Space Power-Sources, School of Chemistry and Chemical Engineering, Harbin Institute of Technology, Harbin 150001, P. R. China

^2^ Key Laboratory for Liquid-Solid Structural Evolution and Processing of Materials, Ministry of Education, Shandong University, Jinan 250061, P. R. China

*Corresponding author. E-mail: [yunchendu@hit.edu.cn](mailto:yunchendu@hit.edu.cn) (Yunchen Du)

**S1 Configurational entropy**

The configurational entropy of the high-entropy alloy is calculated according to the ideal mixing model:

$$\Delta S_{\mathrm{conf}}=-R\sum_{i=1}^{N} x_{i}\ln x_{i} (S1)$$

where *R* denotes the gas constant (8.314 J mol^-1^K^-1^), *x_i_* represents the molar fraction of each constituent element, and N denotes the number of elements involved.

**S2 Electromagnetic wave absorption theory and key parameters**

Based on transmission line theory, the input impedance *Z*_in_*​* at the air–material interface for an absorbing layer of thickness *d* under normal wave incidence is expressed as:

$${\begin{aligned} Z \end{aligned}}_{\mathrm{in}}=Z_{0}\left( \frac{\mu_{r}}{\varepsilon_{r}} \right)^{0.5}\tanh\left[ j\left( \frac{2\pi fd}{c} \right)\left( \mu_{r}\varepsilon_{r} \right)^{0.5} \right] (S2)$$

where *Z*_0_ is the intrinsic impedance of free space, *μ*_r_*=μ*_r_*′-jμ*_r_*′′*and*ε*_r_*=ε*_r_*′-jε*_r_*′′*are the relative complex permeability and permittivity, respectively, *f* is the frequency, and *c* is the speed of light in vacuum.

The reflection loss (RL), which quantifies the ratio of the reflected power to the incident power, is derived from the impedance relationship:

$$\mathrm{RL}=20\lg\left| \frac{Z_{\mathrm{in}}-Z_{0}}{Z_{\mathrm{in}}+Z_{0}} \right| (S3)$$

The impedance matching degree *Z* evaluates the congruence between the material's input impedance and free space impedance:

$$Z=\left| \frac{Z_{\mathrm{in}}}{Z_{0}} \right| (S4)$$

The impedance matching (*Z*≈1) minimizes front-surface reflection, promoting wave penetration into the material.

The attenuation constant *α*, which quantifies the spatial decay rate of the wave inside the material, is calculated as:

$$\alpha=\frac{\sqrt{2}\pi f}{c}\times\sqrt{\left( \mu_{r}^{''}\varepsilon_{r}^{''}-\mu_{r}^{'}\varepsilon_{r}^{'} \right)+\sqrt{\left( \mu_{r}^{''}\varepsilon_{r}^{''}-\mu_{r}^{'}\varepsilon_{r}^{'} \right)^{2}+\left( \mu_{r}^{''}\varepsilon_{r}^{'}+\varepsilon_{r}^{''}\mu_{r}^{'} \right)^{2}}} (S5)$$

A higher *α* value indicates a greater intrinsic electromagnetic energy dissipation capability.

The complex permittivity *ε*_r_ comprises a real part *ε*_r_*′*, governing energy storage, and an imaginary part *ε*_r_*′′*, representing dielectric loss. Within the Debye relaxation framework, *ε*_r_*′* exhibits frequency dispersion:

$\varepsilon_{r}^{'}=\varepsilon_{\infty}+\frac{{(\varepsilon}_{s}-\varepsilon_{\infty})}{1+\left( \omega\tau\right)^{2}}$(S6)

where *ε*_s_ is the static permittivity, *ε*_∞_ is the high-frequency limit permittivity, *ω*=2*πf* is the angular frequency, and *τ* is the polarization relaxation time.

The total dielectric loss *ε*_r_*′′* is composed of two primary contributions: conduction loss *ε*_c_*′′*and polarization relaxation loss *ε*_p_*′′*​:

$\varepsilon_{r}^{''}=\varepsilon_{c}^{''}+\varepsilon_{p}^{''}=\frac{\sigma^{*}}{\omega\varepsilon_{0}}+\frac{{(\varepsilon}_{s}-\varepsilon_{\infty})\omega\tau}{1+\left( \omega\tau\right)^{2}}$(S7)

*σ* denotes the effective electrical conductivity and *ε*_0_ ​is the vacuum permittivity. Thus, the two loss components are explicitly defined as:

$\varepsilon_{c}^{''}=\frac{\sigma}{\pi\varepsilon_{0}f}$(S8)

$\varepsilon_{p}^{''}=\frac{{(\varepsilon}_{s}-\varepsilon_{\infty})\omega\tau}{1+\left( \omega\tau\right)^{2}}$ (S9)

Under the excitation of an alternating EM field, the magnetic response characteristics of the material are described by the complex permeability, denoted as *µ*_r_. This parameter is defined as the combination of a real part and an imaginary part:

$\mu_{r}=\mu_{r}^{'}+j\mu_{r}^{''}$ (S10)

To quantify the attenuation capability of the material regarding EM waves, the magnetic loss tangent (tan*δ_µ_*) is commonly introduced as a performance indicator:

${\tan\delta}_{\mu}=\frac{\mu_{r}^{''}}{\mu_{r}^{'}}$ (S11)

Magnetic loss typically originates from hysteresis loss, domain wall resonance, natural resonance, and eddy current loss. To distinguish the contributions of these mechanisms-specifically to determine if eddy current loss is the dominant factor-the eddy current coefficient (*C*_0_) is widely utilized as a criterion. According to the skin effect theory, if the magnetic loss results solely from eddy current effects, the relationship between *µ*_r_*ʺ* and *f* follows the equation:

$C_{0}=\mu_{r}^{''}\left( \mu_{r}^{'} \right)^{-2}f^{-1}$ (S12)

**S3 Characterizations**

X-ray diffractometer (XRD, Cu Kα, DMAX-2500PC, Rigaku) was employed to analyze the phase composition. Functional groups were identified by Fourier-transform infrared spectroscopy (FT-IR). Inductively coupled plasma optical emission spectrometer (ICP-OES, OPTIMA 7000/700, PerkinElmer) was applied to determine precise elemental composition ratios. Field-emission scanning electron microscope (FE-SEM, Gemini SEM 500, ZEISS) equipped with an energy dispersive spectrometer (EDS) was used to examine microstructural morphology and elemental distribution. Transmission electron microscope (TEM, Talos F200X G2, Thermo Fisher Scientific) was employed to observe nanoscale lattice structures and defect characteristics. Thermal stability was evaluated using thermogravimetric analysis (TGA; TA Instruments Q500). X-ray photoelectron spectrometer (XPS, ESCALAB 250Xi, Thermo Fisher Scientific) was employed to analyze surface elemental chemical states, with all binding energies referenced to the C 1s peak at 284.8 eV. Magnetic properties were measured at room temperature through hysteresis loop analysis using a vibrating-sample magnetometer (VSM; Lake Shore 7404). For electromagnetic performance evaluation, vector network analyzer (3671G, Ceyear) was employed to measure complex permittivity and permeability in the 1-4 GHz frequency range using coaxial ring specimens prepared by mixing the sample (75 wt%) with paraffin (25 wt%). Corrosion behavior was examined by potentiodynamic polarization in 3.5 wt% NaCl solution using an electrochemical workstation (CHI660E, CH Instruments, China) configured with a standard three-electrode system.

**S4 RCS simulation**

In the simulation model, a square absorbing structure with a side length (a) of 1000 mm is utilized. The absorber layer has a thickness (*d*) of 7.18 mm, backed by a perfect electric conductor (PEC) layer with a thickness (*t*) of 1 mm. A plane wave excitation at a frequency of 1.7 GHz is applied. The radar cross section (RCS) is computed to systematically compare the electromagnetic scattering performance of each sample against that of a pure PEC under identical conditions.

The radar cross section (*σ*), which quantifies the scattering strength of a target, is expressed in units of dB·m^2^ in this study. It is calculated using the following formula:

$\left( \mathrm{dB}m^{2} \right)=10\log\left( \left( 4\pi S/\lambda^{2} \right)\left| E_{s}/E_{i} \right| \right)^{2}$ (S13)

where *S* represents the area of the simulation model, *λ* is the wavelength in free space at the simulation frequency, and *E*ₛ and *E*ᵢ refer to the magnitudes of the scattered and incident electric field intensities, respectively.

**S5 DFT calculations**

The Vienna Ab Initio Package (VASP) was employed to perform all the density functional theory (DFT) calculations within the generalized gradient approximation (GGA) using the Perdew, Burke, and Enzerhof (PBE) formulation. The projected augmented wave (PAW) potentials were applied to describe the ionic cores and take valence electrons into account using a plane wave basis set with a kinetic energy cutoff of 450 eV. Partial occupancies of the Kohn-Sham orbitals were allowed using the Gaussian smearing method and a width of 0.05 eV. The electronic energy was considered self-consistent when the energy change was smaller than 10-4 eV. A geometry optimization was considered convergent when the force change was smaller than 0.05 eV/Å. Grimme’s DFT-D3 methodology was used to describe the dispersion interactions. The vacuum spacing perpendicular to the plane of the structure is 20 Å. AIMD simulation was carried out to study structures. During geometry optimization, the cut-off energy was set as 450eV for structures, respectively. And the structures were made by allowing the relaxations of only half of the atoms in the supercells. The Brillouin zone was sampled by a Monkhorst-Pack (MP) k-point grid of 1× 1 × 1 for geometry optimizations. The PBE-D3 dispersion term was introduced to correct the van der Waals interactions. The conjugated gradient method was applied with a smearing width of 0.1 eV, and the convergence criteria for the energy and force were 10-5 eV/cell and 0.05 eV/Å, respectively. AIMD simulations were run for 20 ps as equilibration with time steps of 1 fs, performing a constant temperature of 400 K in the Nosé -Hoover isokinetic ensemble.

**S6 Electrochemical corrosion testing**

The electrochemical corrosion behavior of the materials was evaluated using a three-electrode system. The working electrodes were prepared by coating steel substrates with a composite material, in which SM, LEA, MEA, or HEA was mixed with epoxy resin at a mass ratio of 60:40. A platinum plate and a saturated calomel electrode were used as the counter electrode and reference electrode, respectively. Electrochemical impedance spectroscopy (EIS) and Tafel polarization curves were measured to assess the corrosion resistance performance of the samples.

**Supplementary Figures and Tables**


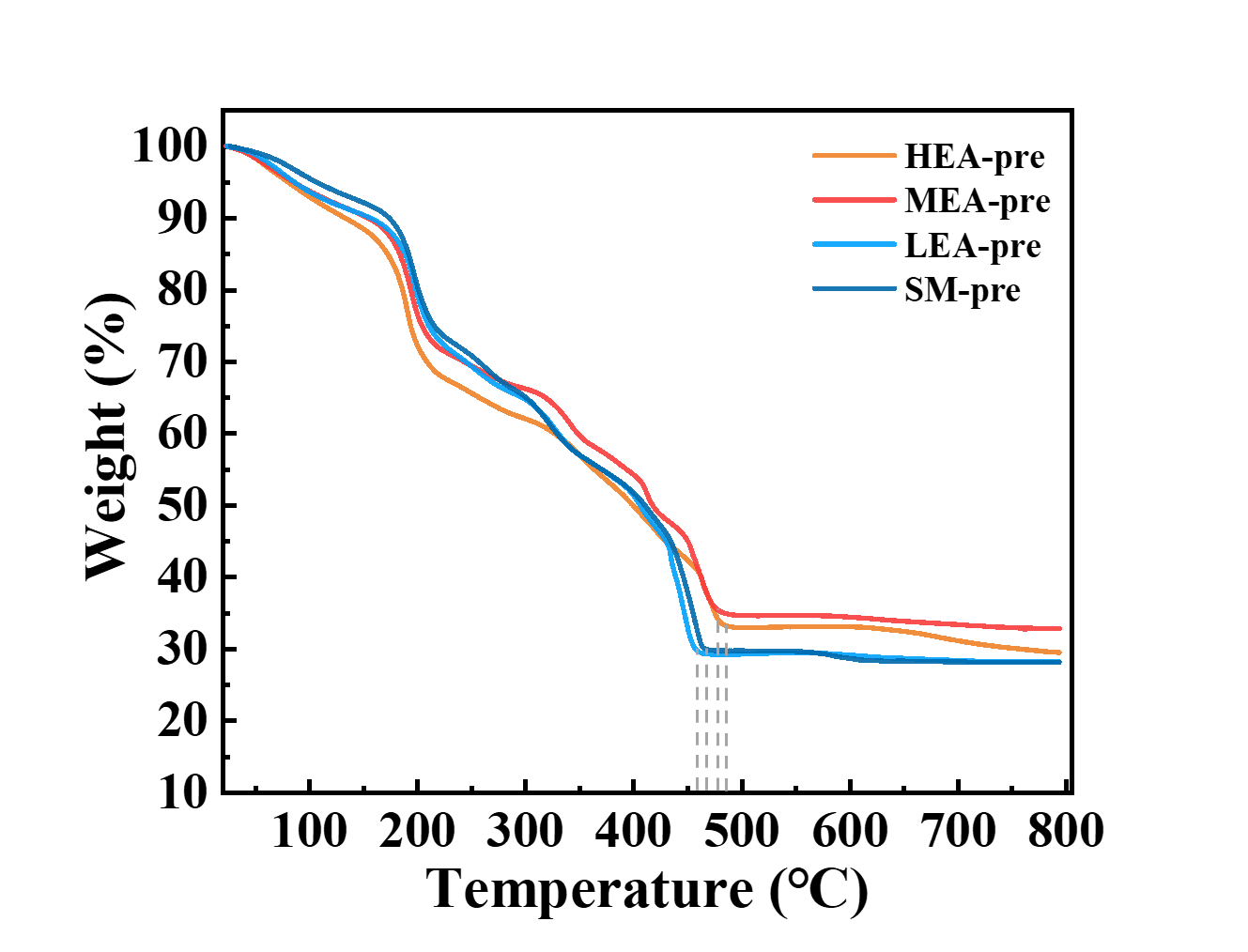


**Fig. S1** TG curve of the precursors

**T****able S1** Molar percentage of elements and configurational entropy in LEA, MEA, and HEA

| MPEAs sample | Fe (%) | Co (%) | Ni (%) | Cr (%) | Cu (%) | Δ*S*_conf_ (R) |
| --- | --- | --- | --- | --- | --- | --- |
| LEA | 86.55 | 4.95 | 5.43 | 1.13 | 1.94 | 0.559 |
| MEA | 62.09 | 13.31 | 13.95 | 5.81 | 4.84 | 1.152 |
| HEA | 33.72 | 22.23 | 23.48 | 11.27 | 9.30 | 1.508 |

**Table S2** BCC and FCC contents in XRD patterns of LEA, MEA, and HEA

| MPEAs sample | BCC | FCC |
| --- | --- | --- |
| LEA | 91.44% | 8.56% |
| MEA | 47.83% | 52.17% |
| HEA | 7.87% | 92.13% |


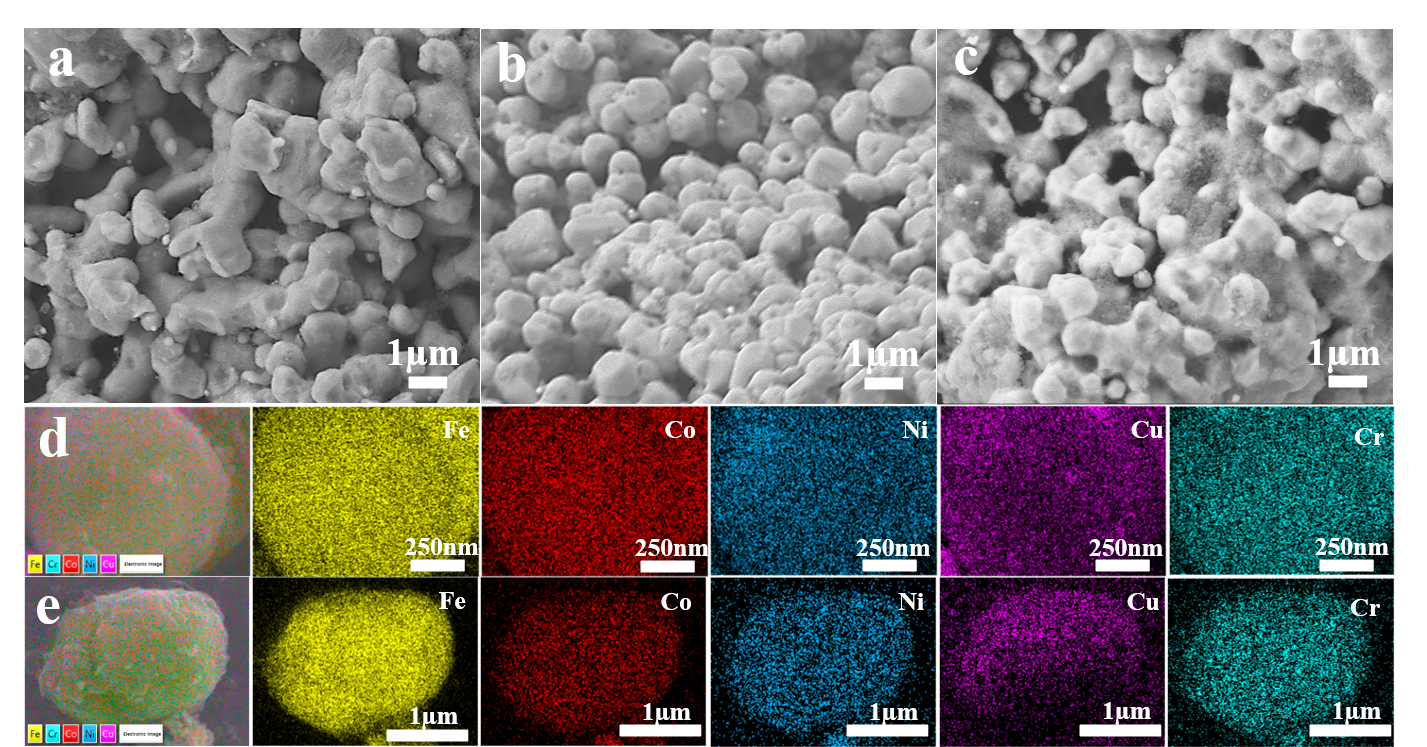


**Fig. S2** The FESEM images of **a** MEA, **b** LEA and **c** SM. EDS elemental mapping of Fe, Co, Ni, Cu, and Cr in **d** MEA and **e** LEA samples


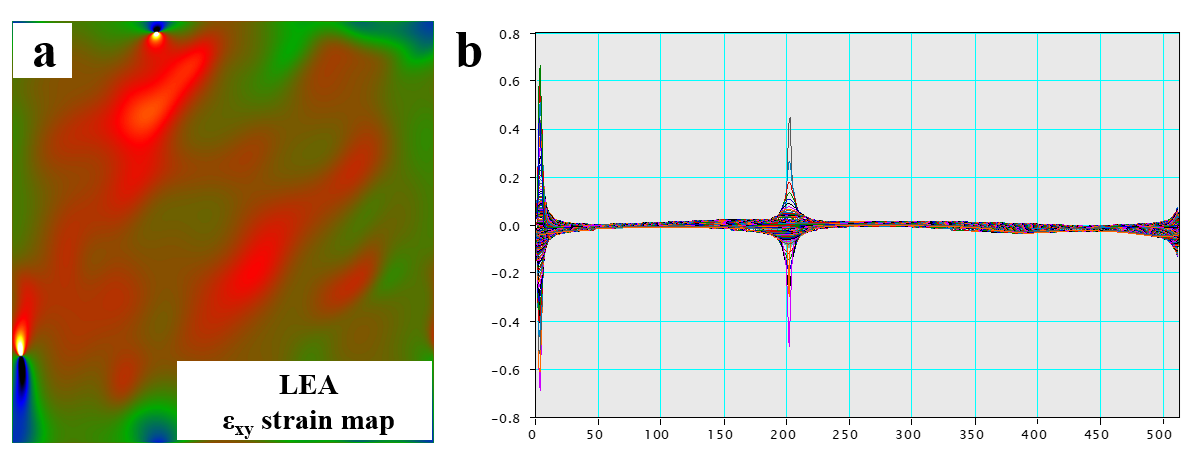


**Fig. S3** **a-b** GPA strain maps of LEA and the corresponding local strain intensity curves


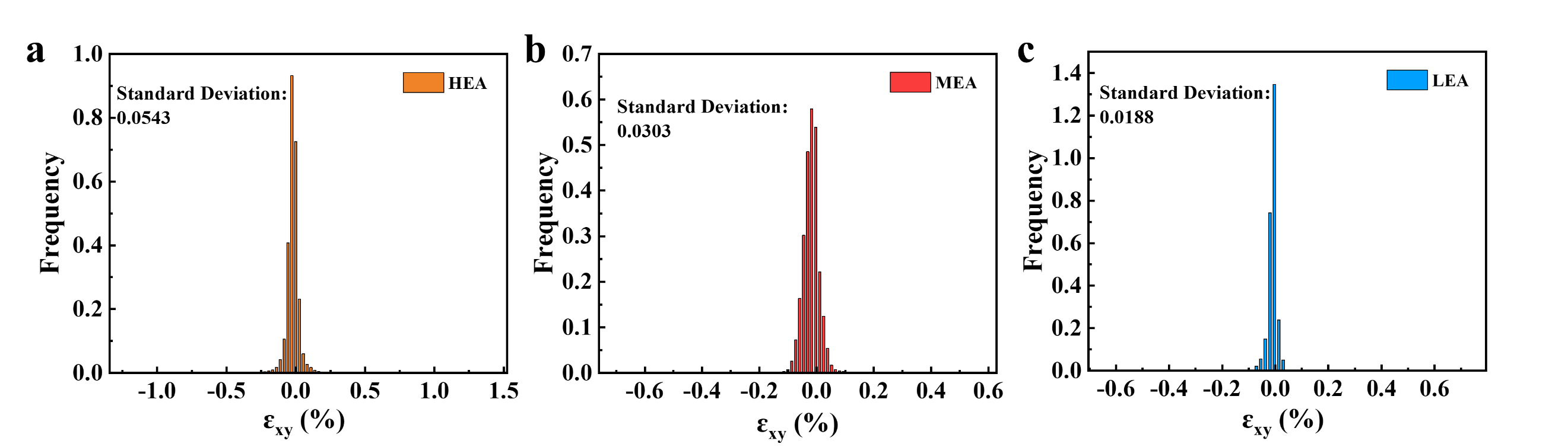


**Fig. S4** **a-c** The corresponding strain distribution images along *ε*_xy_ directions of HEA, MEA, LEA

**Table S3** The comparison in RLmin and EABmax with those reported high-entropy alloy absorbers

| Absorbers | EABmax  (range, thickness) | RLmin  (Frequency, thickness) | Ref. |
| --- | --- | --- | --- |
| FeCoNiCuAl | 2.9 GHz  (7.4-10.2 GHz, 2.63 mm) | -56.1 dB  (6.9 GHz, 3.23 mm) | [36] |
| LIG/HEA-2 | 5.0 GHz  (9.0-14.0 GHz, 3.40 mm) | -41.2 dB  (12.9 GHz, 3.40 mm) | [37] |
| FeCoNiCuM_0.06_ | 3.2 GHz  (7.2-10.5 GHz, 3.05 mm) | -61.8 dB  (10.7 GHz, 3.05 mm) | [38] |
| HCNF/HEA | 7.6 GHz  (10.0-17.6 GHz, 3.00 mm) | -65.8 dB  (11.0 GHz, 3.50 mm) | [39] |
| FeCoNiMnV_2_ | 3.3 GHz  (7.6-10.9 GHz, 2.18 mm) | -51.0 dB  (9.5 GHz, 2.18 mm) | [40] |
| FeCoNiCuTi_0.2_ | 4.7 GHz  (6.0-10.7 GHz, 2.60 mm) | -47.8 dB  (10.8 GHz, 2.16 mm) | [41] |
| FeCoNiCr_0.4_Mn_0.6_ | 5.4 GHz  (8.7-14.1 GHz, 2.00 mm) | -48.5 dB  (6.4 GHz, 3.00 mm) | [42] |
| FeCoNiCuS_0.2_ | 7.0 GHz  (8.0 GHz-15.0 GHz, 2.16 mm) | -55.4 dB  (6.5 GHz, 3.60 mm) | [43] |
| FeCoNiCrAl_0.8_ | 4.7 GHz  (8.7-13.4 GHz, 2.30 mm) | -41.8 dB  (11.9 GHz, 2.30 mm) | [44] |
| FeCoNiMn HEAs | 4.1 GHz  (7.1-11.2 GHz, 2.70 mm) | -62.4 dB  (10.7 GHz, 2.40 mm) | [45] |
| FeCoNiCuCr | 4.5 GHz  (7.2 -11.7 GHz, 2.60 mm) | -41.2 dB  (6.4 GHz, 3.50 mm) | [46] |
| HEO/RGO | 4.1 GHz  (10.8-14.9 GHz 2.50 mm) | -57.1 dB  (14.1 GHz, 2.20 mm) | [47] |
| HEA-This work | 805 MHz  (1.195-2.00 GHz 7.80 mm) | -22.7 dB  (1.7 GHz,7.18 mm) | [This work] |


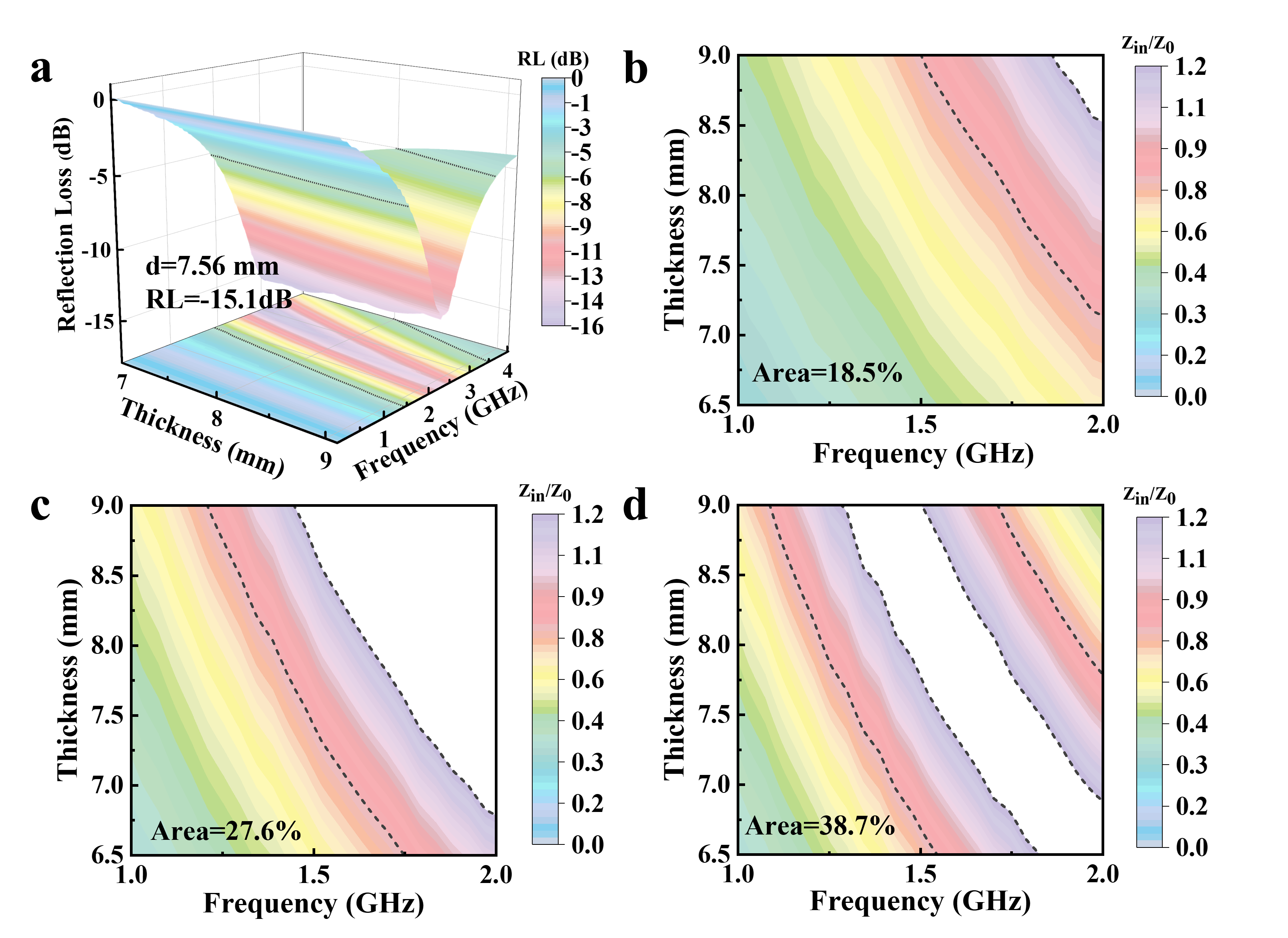


**Fig. S5** **a** 3D diagrams of the reflection loss curve of SM. 2D representations of |Zin/Z0| at different thickness of **b** SM, **c** LEA, **d** MEA


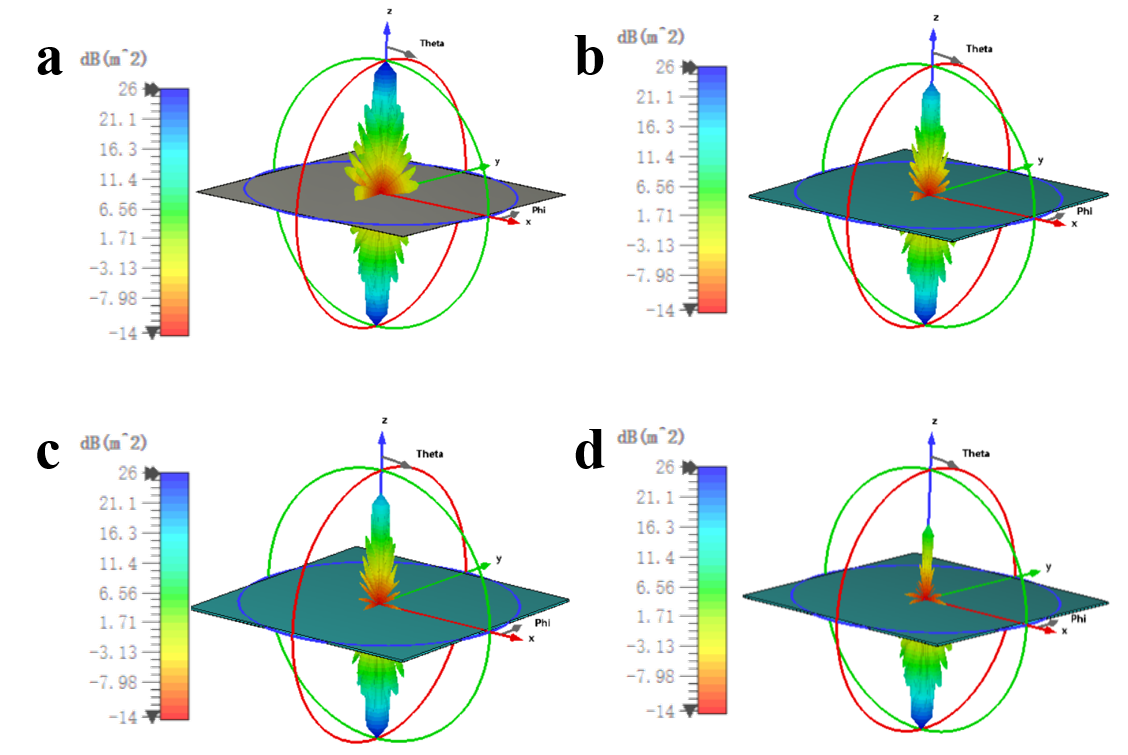


**Fig. S6** **a** 3D RCS plots for the PEC substrate and the PEC substrate covered with **b** SM, **c** LEA, **d** MEA


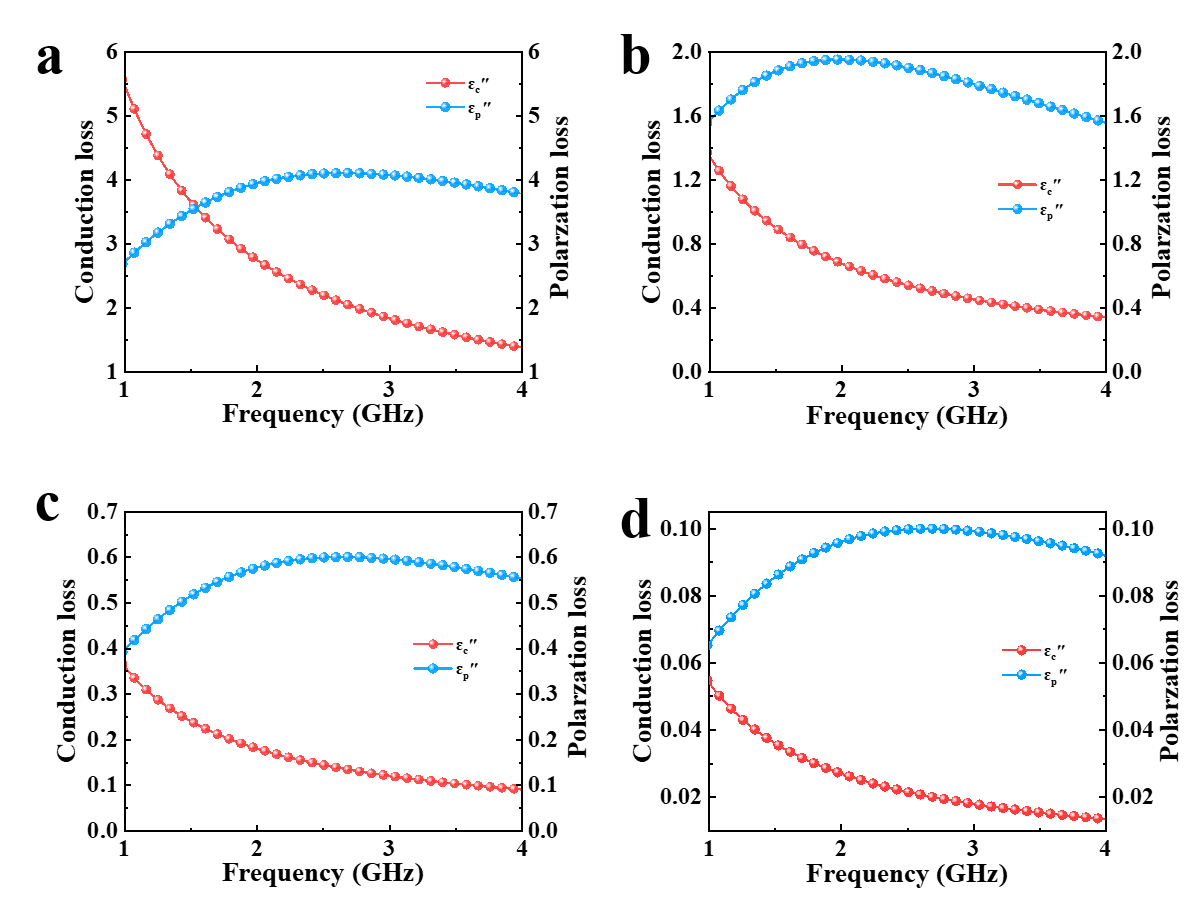


**Fig. S7** Comparison of polarization loss and conduction loss for **a** HEA, **b** MEA, **c** LEA, and **d** SM


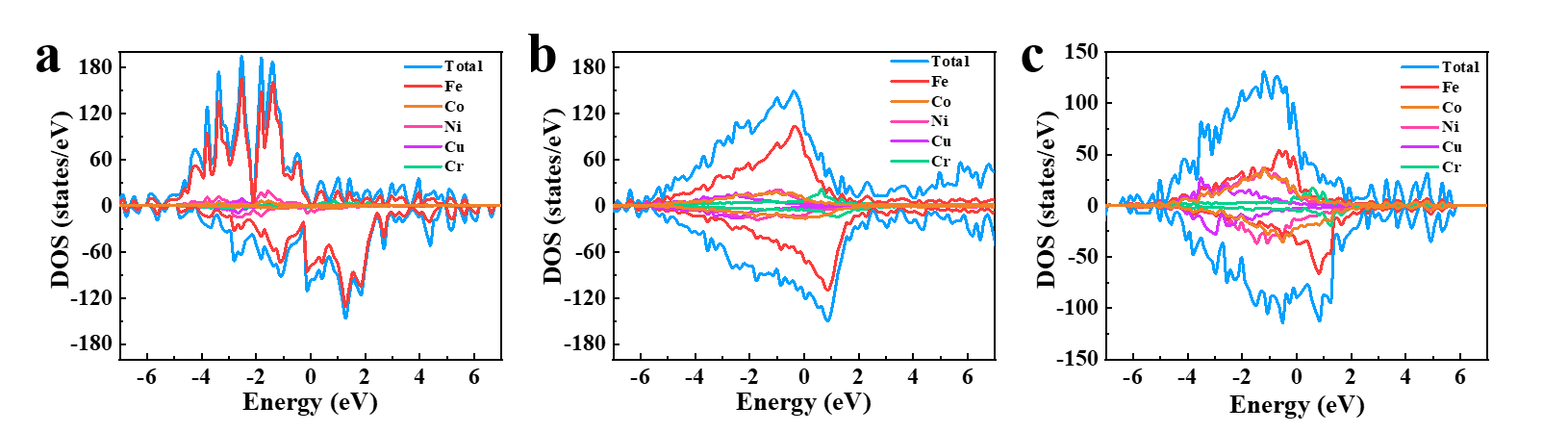


**Fig. S8** Total density of states (DOS) plots of **a** LEA, **b** MEA, and **c** HEA
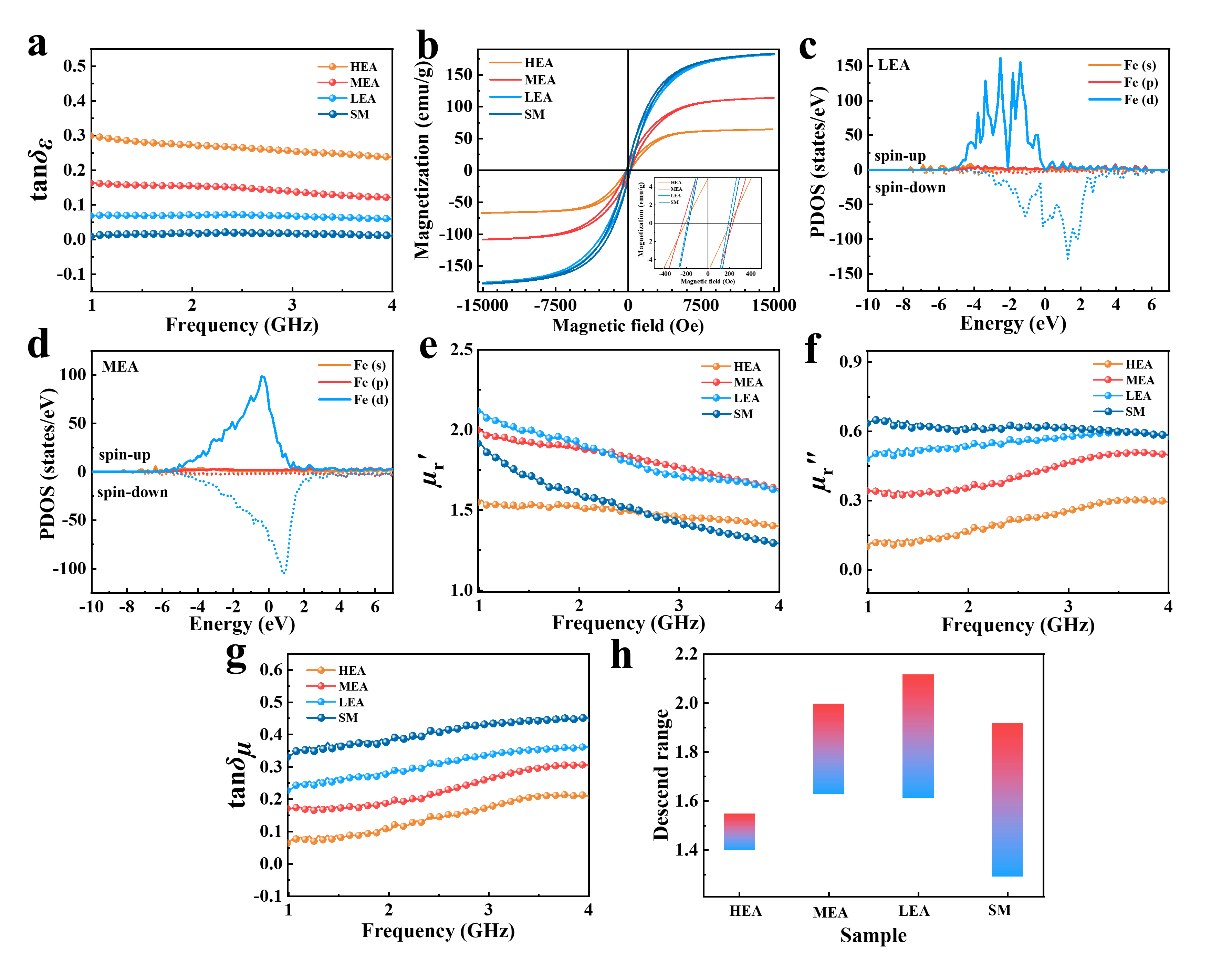


**Fig. S9** **a** Dielectric loss tangent of SM, LEA, MEA, and HEA. **b** Hysteresis loops of SM, LEA, MEA, and HEA. Spin projected density of states (PDOS) based on the ideal model of **c** LEA and **d** MEA. **e-g** Real permeability, imaginary permeability, and magnetic loss tangent of SM, LEA, MEA, and HEA. **h** Reduction range of the real permeability of SM, LEA, MEA and HEA


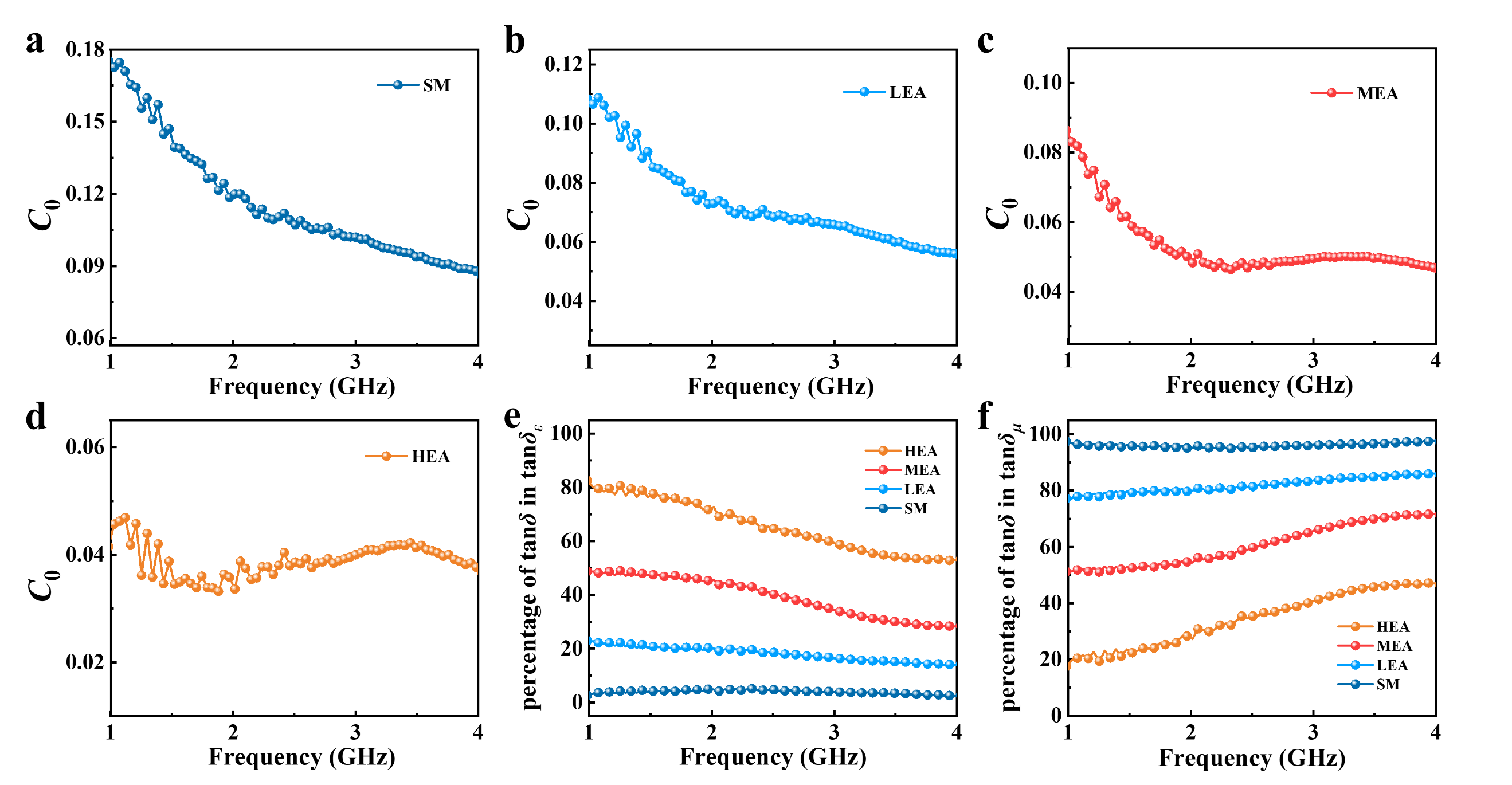


**Fig. S10** Eddy current coefficient (*C*_0_) of **a** SM, **b** LEA, **c** MEA and **d** HEA. The percentage contribution of **e** tan*δ_ε_* and **f** tan*δ_μ_* to the total loss tangent for the samples


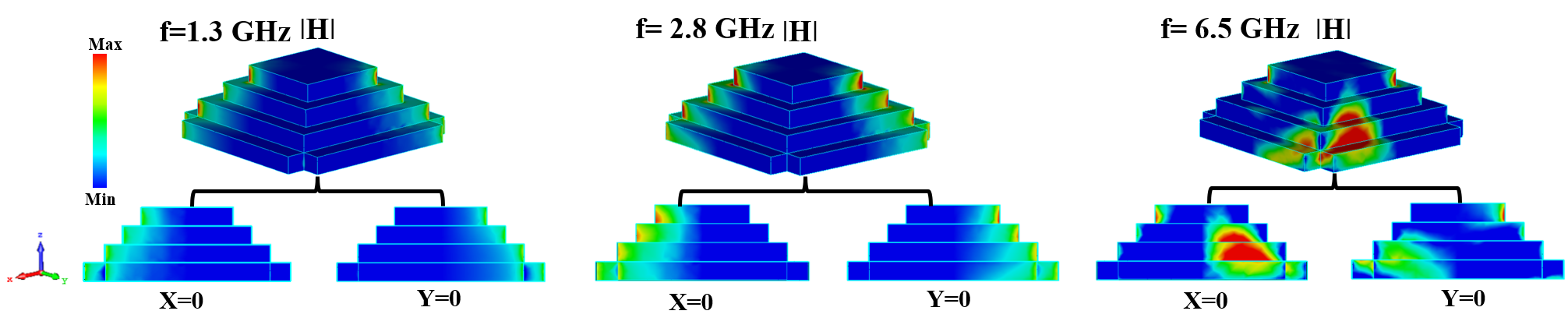


**Fig. S11** Distribution of the magnetic field at 1.3 GHz, 2.8 GHz, and 6.5 GHz for the metamaterial absorber


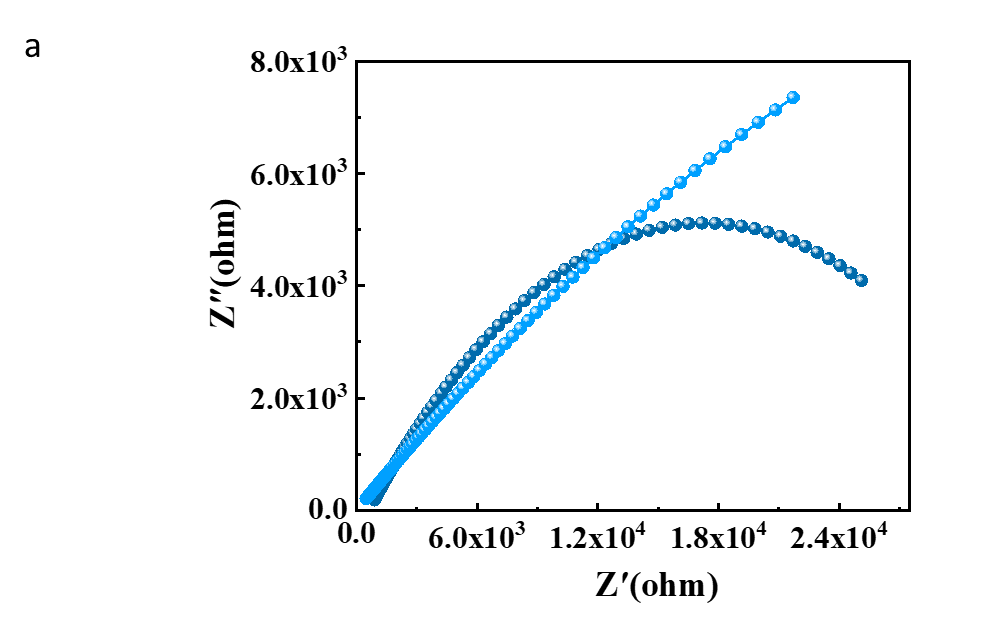


**Fig. S12** Enlarged Nyquist plot of SM and LEA within the dashed box
